# Accurate prediction of cell type-specific transcription factor binding

**DOI:** 10.1186/s13059-018-1614-y

**Published:** 2019-01-10

**Authors:** Jens Keilwagen, Stefan Posch, Jan Grau

**Affiliations:** 1 Institute for Biosafety in Plant Biotechnology, Julius Kühn-Institut (JKI) - Federal Research Centre for Cultivated Plants, Erwin-Baur-Straße 27, Quedlinburg, 06484 Germany; 2 Institute of Computer Science, Martin Luther University Halle–Wittenberg, Von-Seckendorff-Platz 1, Halle (Saale), 06120 Germany

**Keywords:** Transcription factors, DNase-seq, ChIP-seq, Cell type-specific, Machine learning

## Abstract

Prediction of cell type-specific, in vivo transcription factor binding sites is one of the central challenges in regulatory genomics. Here, we present our approach that earned a shared first rank in the “ENCODE-DREAM in vivo Transcription Factor Binding Site Prediction Challenge” in 2017. In post-challenge analyses, we benchmark the influence of different feature sets and find that chromatin accessibility and binding motifs are sufficient to yield state-of-the-art performance. Finally, we provide 682 lists of predicted peaks for a total of 31 transcription factors in 22 primary cell types and tissues and a user-friendly version of our approach, Catchitt, for download.

**Electronic supplementary material:**

The online version of this article (10.1186/s13059-018-1614-y) contains supplementary material, which is available to authorized users.

## Introduction

Activation or repression of transcription is one of the fundamental levels of gene regulation. Transcriptional gene regulation depends on transcription factors (TFs), which specifically bind directly to sites in promoters or enhancers of regulated genes or bind indirectly via other, sequence-specific TFs. Modeling binding specificities, typically represented as sequence motifs, has been an important topic of bioinformatics since its infancy [1, 2]. However, it soon became evident that in silico binding site predictions based on sequence motifs alone are insufficient to explain in vivo binding of TFs and that determinants beyond sequence specificity likely play an important role [3, 4].

The emergence of high-throughput techniques like ChIP-chip [5] or ChIP-seq [6] allowed for experimentally determining in vivo TF binding regions on a genome-wide scale. While especially ChIP-seq and derived techniques have the potential to measure TF-specific and cell type-specific binding, the experimental effort and costs currently preclude ChIP-seq experiments for hundreds to thousands of TFs in a variety of different cell types and tissues. Hence, there is a demand for computational methods predicting cell type-specific TF binding with high accuracy. Fortunately, the existence of genome-wide ChIP data for a subset of TFs and cell types also opens up the opportunity to generate more accurate models by supervised machine learning techniques, which may consider further features beyond motif matches. The main idea of replacing laborious and expensive wet-lab experiments by computational predictions to yield information about further cell types has also been investigated for other epigenomic assays [7, 8].

High-throughput sequencing may also be used to obtain genome-wide assays of chromatin accessibility (e.g., DNase-seq [9] and ATAC-seq [10]), which has been considered one of the key features distinguishing functional from non-functional TF binding sites [11, 12]. Chromatin accessibility data may yield genome-wide maps of functional binding sites of a large fraction of TFs but, in contrast to ChIP-seq, does not identify the TF binding to a specific region. Hence, the association between bound regions (“footprints”) and TFs is typically derived computationally [13].

Following this path, a plenitude of tools (Additional file 1: Table S1; detailed discussion in Additional file 1: Text S1) has been proposed over the last years (e.g., [13–28]). While the general notion of combining sequence signals with chromatin accessibility data and, in some cases, other features is common to the majority of approaches, they differ in several aspects. Specifically, approaches differ in the source of motif information, which may stem from motif databases or from de novo motif discovery. Matches to these motifs are either used as prior information and filtered by their respective DNase-seq signals in a subsequent step, or DNase footprints are first detected and annotated with TFs based on motif matches in those footprints, or, finally, motif and DNase-seq information are processed jointly. Supervised approaches rely on labeled training data, whereas unsupervised approaches may be applied without any a priorily known binding sites of the TF at hand. Finally, motif and chromatin accessibility data may be complemented with further experimental or computational assays like histone modifications or sequence conservation.

Each of these approaches has its benefits and downsides, and the results of benchmark studies in the respective original publications are ambiguous with regard to their prediction performance. Against this background, the “ENCODE-DREAM in vivo Transcription Factor Binding Site Prediction Challenge” (https://www.synapse.org/#!Synapse:syn6131484) aimed at assessing the performance of tools for predicting cell type-specific TF binding in human using a minimal set of experimental data in a fair and unbiased manner. The challenge setting has advantages over typical benchmark studies, because approaches are typically applied to the challenge data by their authors, ground truth is known only by the challenge organizers, and participants are typically required to provide code and documentation for their method such that predictions can be reproduced.

Participants in the ENCODE-DREAM challenge were allowed to use binding motifs from any source, genomic sequence, gene annotations, in silico DNA shape predictions, and cell type-specific DNase-seq and RNA-seq data. In addition, TF ChIP-seq data and ChIP-seq-derived labels (“bound,” “unbound,” “ambiguous”) were provided for training cell types and training chromosomes. Predictions had then to be made for combinations of TF and cell type not present in the training data on held-out chromosomes. Predictions were evaluated against labels derived from TF ChIP-seq data for that specific TF and test cell type.

Here, we present our approach for predicting cell type-specific TF binding regions earning a shared first rank among 40 international teams, including developers of several established methods (cf. Additional file 1: Tables S2 and S3). For our approach, the AUC-PR on unseen test data of a new cell type varies between 0.25 and 0.81 with a median 0.41. From a practical perspective, this means that even state-of-the-art computational approaches are currently not on a level of accuracy that would allow for replacing wet-lab experiments like ChIP-seq.

The approach presented in this paper combines several novel ideas. First, we consider motifs from databases, but also motifs learned by de novo motif discovery from ChIP-seq and DNase-seq data using sparse local inhomogeneous mixture (Slim) models [29], which may capture short- to mid-range intra-motif dependencies. Second, we process DNase-seq data following the binning idea of previous approaches but defining novel statistics computed from the data in those bins, and derive several sequence-based, annotation-based, and RNA-seq-based features. Third, we apply a supervised machine learning approach that employs a discriminative learning principle, which is related to logistic regression but allows for explicit model assumptions with regard to different features. Fourth, discriminative learning is combined with an iterative training approach for refining sets of representative negative examples. Finally, we combine the predictions of classifiers trained in different of these iterations and on different training cell types in an ensemble-like approach.

As this novel approach has already been benchmarked against a large number of competing approaches as part of the ENCODE-DREAM challenge (https://www.synapse.org/#!Synapse:syn6131484/wiki/405275), we focus on the analysis for the contributions of different aspects of this approach on the final prediction performance in this paper. Specifically, we evaluate the contribution of different features, we compare the performance achieved by standard training with that achieved by the iterative training procedure, and we assess the performance of individual classifiers compared with their ensemble prediction. Based on these analyses, we define and benchmark a simplified variant of the proposed approach. Finally, we provide a large collection of predicted, cell type-specific tracks of binding regions for 31 TFs in 22 primary cell types and tissues to make predictions by this approach readily accessible.

## Results

During the ENCODE-DREAM challenge, a large number of approaches created by 40 international teams have been benchmarked on 13 cell type-specific ChIP-seq assays for 12 different TFs in human (Additional file 1: Figure S1). A set of 109 data sets for the same (and additional) TFs in other cell types was provided for training. Training data comprised cell type-specific DNase-seq data, cell type-specific RNA-seq data, genomic sequence and annotations, and in silico DNA shape predictions. In addition, cell type-specific and TF-specific ChIP-seq data and derived labels were provided for training chromosomes, while predictions were evaluated only on the remaining, held-out chromosomes chr1, chr8, and chr21 that were not provided with any of the ChIP-seq training data. For 200-bp regions shifted by 50 bp, genome-wide predictions of the probability that a specific region overlaps a ChIP-seq peak were requested from the participating teams. Predictions were evaluated by (i) the area under the ROC curve (AUC-ROC), (ii) the area under the precision-recall curve (AUC-PR), (iii) recall at 10% FDR, and (iv) recall at 50% FDR on each of the 13 test data sets. These were aggregated per data set based on the average, normalized rank earned for each of these measures in 10 bootstrap samples of the held-out chromosomes, and a final ranking was obtained as the average of these rank statistics (cf. https://www.synapse.org/#!Synapse:syn6131484/wiki/405275).

As a result of this ranking, the approach presented in this paper (team “J-Team”) earned a shared first rank together with the approach created by team “Yuanfang Guan.”

In the following, we investigate the influence of different aspects of the proposed approach on the final prediction performance. First, we inspect the impact of different sets of related features (DNase-seq data, motif scores, RNA-seq data, sequence-based and annotation-based features) on prediction performance. Second, we study the importance of the iterative training approach as opposed to a training on initial training data. Third, we compare the performance of the predictions gained by classifiers trained on training data for individual cell types with the performance of the aggregated prediction obtained by averaging over these cell types. Finally, we apply the proposed method for predicting cell type-specific TF binding for 31 TFs in 22 additional primary cell types yielding a total of 682 prediction tracks.

### Impact of feature sets on prediction performance

**Fig. 1:**
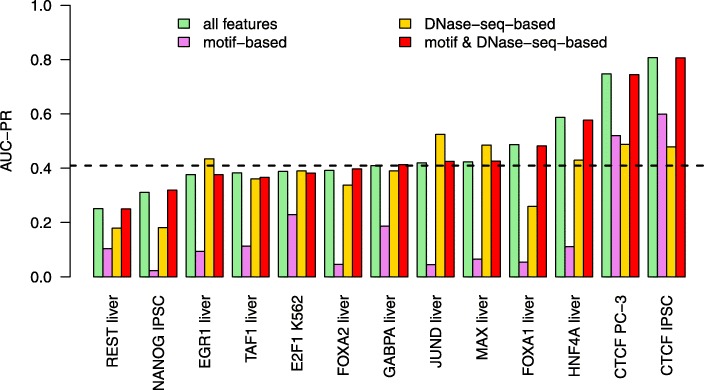
Across cell type performance. For each of the 13 combinations of TF and cell type within the test data, we compute the prediction performance (AUC-PR) on the held-out chromosomes of classifiers (i) using all features considered, (ii) using only motif-based features, (iii) using only DNase-seq-based features, and (iv) using only motif-based and DNase-seq-based features. Median performance of classifiers using all features is indicated by a dashed line

We use the prediction performance obtained by the proposed approach using all sets of features (“Features” section), the iterative training procedure (“Iterative training” section), and the aggregation over all training cell types (“Prediction schema” section) as a baseline for all further comparisons (Fig. 1; “all features”). Throughout this manuscript, we consider AUC-PR as the primary performance measure, since AUC-PR is more informative about classification performance for heavily imbalanced classification problems [30, 31], and recall at the different FDR levels is rather unstable since it corresponds to single points on the precision-recall curve. AUC-PR values are computed using the R-package PRROC [32], which has also been used in the ENCODE-DREAM challenge.

We find that prediction performance as measured by AUC-PR varies greatly among the different transcription factors (Fig. 1) with a median AUC-PR value of 0.4098. The best prediction performance is achieved for CTCF, which has a long and information-rich binding motif, in two different cell types (IPSC and PC-3). Above-average performance is also obtained for FOXA1 and HNF4A in liver cells. For most other TFs, we find AUC-PR values around 0.4, whereas we observe a rather low prediction accuracy for NANOG and REST.

**Fig. 2:**
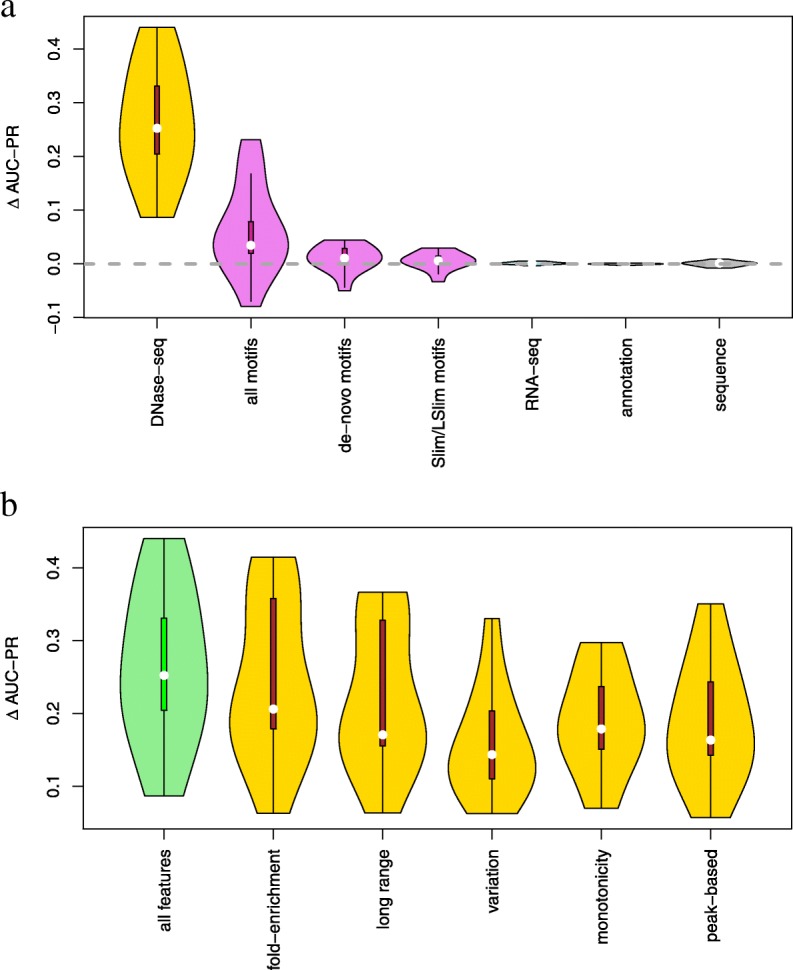
Importance of feature sets. **a** We test the importance of related sets of features by excluding one set of features from the training data, measuring the performance (AUC-PR) of the resulting classifier, and subtracting this AUC-PR value from the corresponding value achieved by the classifier using all features. Hence, if *Δ* AUC-PR is above zero, the left-out set of features improved the final prediction performance, whereas *Δ* AUC-PR values below zero indicate a negative effect on prediction performance. We collect the *Δ* AUC-PR values for all 13 test data sets and visualize these as violin plots. **b** Assessment of different groups of DNase-seq-based features. In this case, we compare the performance including one specific group of DNase-seq-based features (cf. Additional file 1: Text S2)) with the performance without any DNase-seq-based features (cf. violin “DNase-seq” in panel **a**). We find that all DNase-seq-based features contribute positively to prediction performance

To analyze the contribution of selected features on the final prediction performance, we systematically exclude sets of related features from the input data in training and prediction. As a baseline, we measure AUC-PR for the classifier using all feature sets. In addition, we measure AUC-PR when excluding each individual feature set, where the difference of these two AUC-PR values quantifies the improvement gained by including the feature set (Fig. 2a).

We observe the greatest impact for the set of features derived from DNase-seq data. The improvement in AUC-PR gained by including DNase-seq data varies between 0.087 for E2F1 and 0.440 for HNF4A with a median of 0.252.

Features based on motif scores (including de novo discovered motifs and those from databases) also contribute substantially to the final prediction performance. Here, we observe large improvements for some TFs, namely 0.231 for CTCF in IPSC cells, 0.175 for CTCF in PC-3 cells, and 0.167 for FOXA1. By contrast, we observe a decrease in prediction performance in the case of JUND (− 0.080) when including motif-based features. For the remaining TFs, we find improvements of AUC-PR between 0.008 and 0.079. We further consider two subsets of motifs, namely all motifs obtained by de novo motif discovery on the challenge data and all Slim/LSlim models capturing intra-motif dependencies. For motifs from de novo motif discovery, we find an improvement for 9 of the 13 data sets, and for Slim/LSlim model, we find an improvement for 10 of the 13 data sets. However, the absolute improvements (median of 0.011 and 0.006, respectively) are rather small, possibly because (i) motifs obtained by de novo motif discovery might be redundant to those found in databases and (ii) intra-motif dependencies and heterogeneities captured by Slim/LSlim models [29] might be partly covered by variations in the motifs from different sources.

Notably, RNA-seq-based features (median 0.001), annotation-based features (0.000), and sequence-based features (0.001) have almost no influence on prediction performance.

As the set of DNase-seq-based features is rather diverse, including features derived from fold-enrichment tracks, peak lists, or variation among cell types, we aim at further dissecting the influence of related groups of those features. To this end, we further test how prediction performance is affected by removing specific groups of DNase-seq features (cf. Additional file 1: Text S2) from the complete feature set (Additional file 1: Figure S2). Notably, we find that none of these feature groups alone have a large impact on prediction performance, although gradual differences may be observed as the inclusion of fold-enrichment-based and peak-based features have a largely positive contribution, whereas the influence of the other feature groups is rather ambiguous. This might be explained by wide redundancies and correlations that still exist between those different groups, which allows for large compensation for the loss of a single feature group.

Hence, we additionally test a scenario, where leaving out all DNase-seq-based features (i.e., the data behind the violin plot “DNase-seq” of Fig. 2a) is considered the baseline case, and only one of the specific groups is added to this reduced feature set (Fig. 2b). First of all, we observe that all feature groups contribute positively to the total prediction performance. The largest contribution may be observed for the “fold-enrichment” group, but also related groups like “long range” basically averaging over broader windows of the fold-enrichment track, and “peak-based” using peaks that have originally been called based on DNase-seq coverage. We find the lowest contribution for the “variation” group, which measures the variation and conservation, respectively, of the DNase-seq signal among the cell types. As the contribution of each individual group of features is positive, we still consider the complete set of DNase-seq-based features in the following.

Having established that DNase-seq-based and motif-based features have a large impact on prediction performance, we also tested the prediction performance of the proposed approach using *only* features based on DNase-seq data and TF motifs, respectively. All other features, i.e., RNA-seq-based features, annotation-based features, and features based on raw sequence, are excluded. We find (Fig. 1) that classifiers using exclusively motif-based features already yield a reasonable prediction performance for some TFs (CTCF and, to some extent, E2F1 and GABPA), whereas we observe AUC-PR values below 0.12 for the remaining of TFs. This may be explained by the large number of false positive predictions typically generated by approaches using exclusively motif information, which may only be avoided in case of long, specific motifs as it is the case for CTCF.

Classifiers using only DNase-seq-based features yield a remarkable performance for many of the TFs studied (Fig. 1), which is lower than for the motif-based classifier only for the two CTCF datasets. For some datasets (especially JUND but also EGR1, MAX), we even observe that a classifier based on DNase-seq data alone outperforms the classifier utilizing all features.

In the case of JUND, the increase in performance when neglecting all non-DNase features can likely be attributed to a strong adaptation of classifier parameters to either cell type-specific binding motifs or cell type-specific co-binding with other TFs, because JUND is the only dataset with an improved performance when excluding motif-based features as discussed above. For all three TFs, we do find an improvement of prediction performance if classifier parameters are trained on the training chromosomes of the test cell type (“within cell type” case; Additional file 1: Figure S3).

Since DNase-seq-based and motif-based features appear to be the primary feature sets affecting prediction performance, we finally study prediction performance of a classifier using only these two feature sets. We observe that prediction performance using only DNase-seq-based and motif-based features is largely identical to that of the classifier using all features (Fig. 1), where we observe the largest loss in AUC-PR for TAF1 (0.017) and the largest gain in AUC-PR for NANOG (0.007). We notice a similar behavior for the within cell type case (Additional file 1: Figure S3). As the left-out feature sets include all RNA-seq-based features, this also has the consequence that one cell type-specific assay (namely DNase-seq) is sufficient for predicting TF binding, which broadens the scope of cell types with readily available experimental data that the proposed approach may be applied to.

### Iterative training improves prediction performance

As a second key aspect of the proposed approach, we investigate the impact of the iterative training procedure on the final prediction performance. To this end, we compare for each TF the AUC-PR values obtained by averaging over the predictions all five classifiers resulting from the iterative training procedure for all training cell types with the AUC-PR values obtained by only averaging over the initially trained classifiers for all training cell types, i.e., classifiers trained only on the initial training data (“Initial training data” section).

**Fig. 3:**
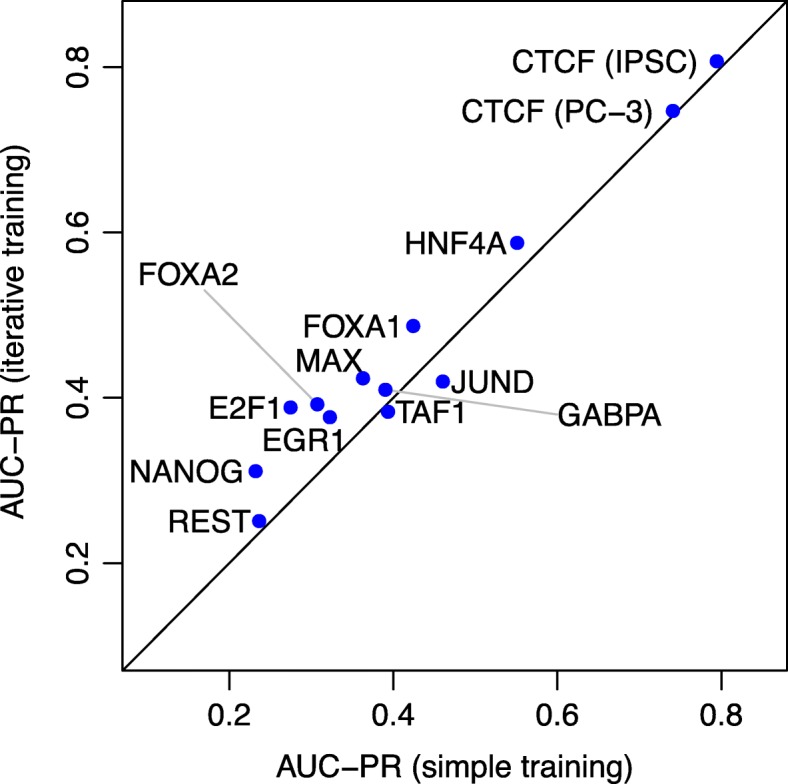
Relevance of the iterative training procedure. For each of the 13 test data sets, we compare the performance (AUC-PR) achieved by the (set of) classifier(s) trained on the initial negative regions (abscissa) with the performance achieved by averaging over all classifiers from the iterative training procedure (ordinate)

For 11 of the 13 test data sets, we observe an improvement of prediction performance by the iterative training procedure (Fig. 3). The largest improvements are achieved for E2F1 (0.114), FOXA2 (0.085), NANOG (0.08), FOXA1 (0.063), and MAX (0.061). Among these are TFs for that we observed a good performance using only DNase-seq-based features (E2F1, MAX) and TFs for which the combination with motif-based features was beneficial (FOXA1, FOXA2, NANOG), which indicates that the additional negative regions added in iterations 2 to 5 do not induce a bias towards either of these two feature types. For four of these five TFs, only one (FOXA2, NANOG, FOXA1) or two (E2F1) training cell types were provided, and the variation between the different classifiers from iterative training may help to avoid overfitting. By contrast, we find a decrease in performance for JUND (0.041) and also TAF1 (0.01), which might be caused by a stronger emphasis on cell type-specific binding regions in subsequent iterations of the iterative training procedure. This hypothesis is also supported by the observation that the iterative training procedure always leads to an increase in prediction performance if classifier parameters are trained on the training chromosomes of the test cell type (Additional file 1: Figure S4).

### Averaging predictions improves over random selection of cell types

For 9 of the 12 TFs considered, data for more than one training cell type is provided with the challenge data. Hence, one central question might be the choice of the cell type used for training and, subsequently, for making predictions for the test cell type. However, the only cell type-specific experimental data available for making that choice are DNase-seq and RNA-seq data, whereas similarity of cell types might depend on the TF considered. Indeed, similarity measures derived from DNase-seq data (e.g., Jaccard coefficients of overlapping DNase-seq peaks, correlation of profiles) or from RNA-seq data (e.g., correlation of TPM values) showed to be non-informative with regard to the similarity of TF binding regions in preliminary studies on the training cell types.

**Fig. 4:**
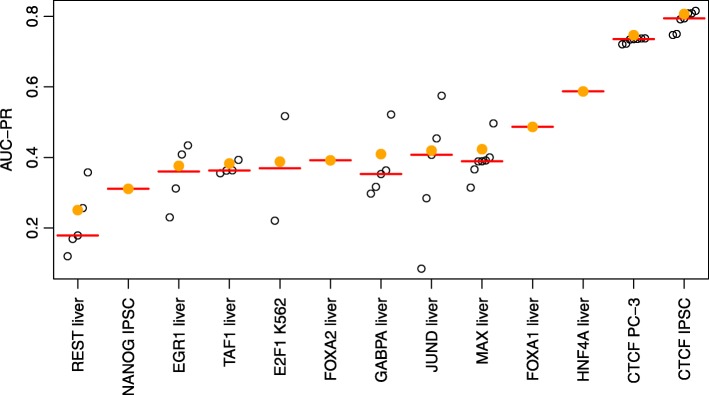
Performance of ensemble classifiers. For each of the 13 test data sets, we compare the performance (AUC-PR) of the individual classifiers trained on single cell types (open circles) to that of the ensemble classifier averaging over all classifiers trained on all training cell types (filled, orange circles). As a reference, we also plot the median of the individual classifiers as a red bar

Hence, we consider the choice of the training cell type a latent variable, and average over the predictions generated by the respective classifiers (see the “Prediction schema” section). As labels of the test cell types have been made available after the challenge, we may now evaluate the impact of this choice on prediction performance and also test the prediction performance of classifiers trained on individual cell types (Fig. 4).

For all test data sets with multiple training cell types available, we find that the averaged prediction yields AUC-PR values above the median of the AUC-PR values achieved for individual training cell types. This improvement is especially pronounced for REST, GABPA, and MAX.

To further investigate if averaging over classifiers for individual cell types favors conserved binding regions (i.e., regions labeled as “bound” in the majority of cell types) over cell type-specific binding regions, we also assess prediction performance on such regions separately (Additional file 1: Figure S5). Specifically, we consider a bound region conserved if it is also labeled as “bound” in at least three of four training cell types, and we consider a bound region as cell type-specific if this region is labeled as “bound” in at most one of four training cell types. The first thing we notice from Additional file 1: Figure S5 is that the absolute AUC-PR values are substantially lower for cell type-specific regions than for conserved regions. One explanation could be a difference in the class (im-)balance due to the selected subsets of regions. However, this general trend remains when considering AUC-ROC (Additional file 1: Figure S6). Second, we find that the variation between classifiers learned from different training cell types is in most cases larger for the cell type-specific regions than for the conserved regions. The behavior with regard to absolute performance is similar for the individual classifiers, their median performance, and the performance of averaging over classifiers for individual cell types. We notice that the AUC-PR gained by averaging is always better than the median performance for individual cell types for conserved regions, but the same holds true when considering cell type-specific regions for seven of the nine data sets with more than one training cell type.

Hence, we may argue that averaging over the cell type-specific classifiers generally yields more accurate predictions than would be achieved by an uninformed choice of one specific training cell type.

However, we also notice for almost all test data sets with multiple training cell types (the only exception being CTCF for the PC-3 cell type) that the best prediction performance achieved for one of the individual training cell types would have gained, in some cases considerable, improvements over the proposed averaging procedure. Notably, the variance of AUC-PR between the different training cell types is especially pronounced for JUND, which supports the previous hypothesis that some features, for instance binding motifs or co-binding of TFs, are highly cell type-specific for JUND. In general, deriving informative measures of TF-specific cell type similarity based on cell type-specific assays and preliminary binding site predictions, would likely lead to a further boost of the performance of computational approaches for predicting cell type-specific TF binding.

### Creating a collection of cell type-specific TF binding tracks

Having established that a single type of experimental assay, namely DNase-seq, is sufficient for predicting cell type-specific TF binding with state-of-the-art accuracy, we may now use the classifiers obtained on the training cell types and TFs for predictions on further cell types. For this purpose, we use the classifiers considering only DNase-seq-based and motif-based features, but neither RNA-seq-based features, annotation-based features, nor features based on raw sequence, which showed to achieve a prediction performance comparable to the full model before (cf. Fig. 1, section “Impact of feature sets of prediction performance”). To this end, we download DNase-seq data for a collection of primary cell types and tissues (see “Data” section), process these in the same manner as the original challenge data, and, subsequently, extract DNase-seq-dependent features (“Features” section). We then applied the trained classifiers for all 31 TFs considered in the challenge to these 22 DNase-seq feature sets to yield a total of 682 prediction tracks.

For the selected cell types (Additional file 1: Table S5), only few cell type and TF-specific ChIP-seq data are available (Additional file 1: Table S6). On the one hand, this means that the predicted TF binding tracks provide valuable, novel information for the collection of 31 TFs studied. On the other hand, this provides the opportunity to perform benchmarking and sanity checks with regard to the predictions for the subset of these TFs and cell types with corresponding ChIP-seq data available. For benchmarking, we additionally obtain the “relaxed” and (where available) “conservative” peak files from ENCODE and derive the associated labels (“bound,” “unbound,” “ambiguous”) according to the procedure proposed for the ENCODE-DREAM challenge.

For CTCF with ChIP-seq peaks available for multiple cell types, we generally find a prediction performance that is comparable to the performance observed on the challenge data (cf. Additional file 1: Table S4). For these cell types, AUC-PR values (Additional file 1: Table S7) range between 0.7720 and 0.8197 if conservative and relaxed peaks are available and if the donors match between the DNase-seq and ChIP-seq experiments, while performance is slightly lower for non-matching donors (0.7322) and in case of missing conservative peaks (0.7270). For JUN, MAX, and MYC, only relaxed peaks are available from ENCODE due to missing replicates. Here, we find AUC-PR values of 0.6310 for JUN, which is substantially larger than for the challenge data; 0.4004 for MAX, which is slightly lower than for the challenge data; and 0.1989 for MYC, which has not been among the test TFs in the challenge but obtained substantially better performance in the leaderboard round.

The 682 genome-wide prediction tracks are still rather large (approx. 880 MB per track) and, hence, demand for substantial storage space that might not be available to the typical user, while the majority of regions are likely not bound by the TF of interest. Hence, we further condense these predictions into predicted peak lists in narrowPeak format by joining contiguous stretches with high binding probability and applying a threshold of 0.6 (relaxed) and 0.8 (conservative) on the maximum probability observed in a predicted “peak.” We provide these peak files for download at https://www.synapse.org/#!Synapse:syn11526239 (doi:10.7303/syn11526239).

To get an impression of the quality of the predicted peaks, we further compute Jaccard coefficients based on peak overlaps (computed using the GenomicRanges R-package [33]) between the predicted peak files and those from the corresponding, available ChIP-seq peaks (Additional file 1: Table S9, S11), and find those to be widely concordant to the previous assessment based on the derived labels.

Finally, the data for CTCF allow for comparing the overlap between predicted peak lists and experimentally determined peak lists to the overlaps observed for (i) technical replicates (Additional file 1: Table S12) and (ii) biological replicates (Additional file 1: Table S10). We find that the overlaps between predictions and IDR-thresholded peaks are lower than those between IDR-thresholded peaks and/or technical replicates. For CTCF, three independent experiments for “foreskin fibroblast” tissue are available, and we use two independent DNase-seq samples for that tissue for our prediction. Comparing the Jaccard coefficients in those two situations (cf. Additional file 1: Tables S9, S10), we find that Jaccard coefficients between predictions and IDR-thresholded peaks vary between 0.568 and 0.693, while we observe Jaccard coefficients between 0.658 and 0.72 for biological replicates. Based on these limited data, we might conclude that computational predictions are less consistent than biological replicates only by a small margin, at least for CTCF.

Based on the predicted peak lists, we may also compare the predicted binding characteristics of the different TFs across cell types. First, we inspect the number of predicted peaks per TF and cell type (Additional file 1: Figure S7). We find a distinct group of highly abundant TFs (CTCF, GATA3, SPI1, CEBPB, FOXA1, FOXA2, MAX), which typically also show large numbers of peaks in the training data. Among these, we find patterns of cell type specificity from the ubiquitously abundant CTCF to largerly varying abundance for GATA3. The remainder of TFs obtains substantially lower numbers of predicted peaks with similar patterns, e.g, for ATF7/ARID3A/NANOG or EP300/TEAD4/JUND, where the latter group has been reported to co-bind in distal enhancers [34]. Next, we study the stability of peak predictions, i.e., the Jaccard coefficients of peaks predicted for each of the TFs in different cell types (Additional file 1: Figure S8). Again, we find substantial variation among the TFs with GABPA, CTCF, and REST having median Jaccard coefficients above 0.7. Notably, CTCF has been one of the TFs with the largest number of predicted peaks (median 37 455), whereas we observed an order of magnitude less predicted peaks for REST (median 3 364) and GABPA (median 5 430). At the other end of the scale, we find indirectly binding TFs like EP300, or TFs that are highly specific to cell types under-represented in our data like NANOG (stem cells) and HNF4A (liver, kidney, intestines). Finally, we investigate co-binding of TFs by computing the average Jaccard coefficient across cell types for each pair of TFs (Additional file 1: Figure S9). Here, we observe distinct groups of co-occurring TFs like CTCF/ZNF143 or FOXA1/FOXA2, which are known to interact in vivo [35–37]. In addition, we find a larger cluster of TFs with substantial overlaps between their predicted peaks comprising YY1, MAX, CREB1, MYC, E2F6, E2F1, and TAF1. As TAF1 (TATA-box binding protein associated factor 1) is associated with transcriptional initiation at the TATA box, one explanation might be that binding sites of these TFs are enriched at core promoters. Indeed, binding to proximal promoters has been reported for MYC/MAX [38], CREB1 [39], YY1 [40], and E2F factors [41].

### Streamlined Catchitt implementation yields competitive performance

We finally compare Catchitt, the simplified implementation of the iterative training approach combining chromatin accessibility and motif scores, to the challenge implementation using DNase-seq-based and motif-based features for the within cell type case. To this end, we select five combinations of cell type and transcription factor spanning the range of performance values observed in the challenge. Specifically, we consider NANOG and TAF1, which obtained the lowest AUC-PR values (cf. Additional file 1: Figure S3) for the challenge implementation; CTCF in IPSC cells, which obtained the largest AUC-PR value; and FOXA1 and HNF4A, which obtained medium AUC-PR values but profited substantially from iterative training (cf. Additional file 1: Figure S4). We summarize the results of this comparison in Additional file 1: Table S13. Despite approximately tenfold reduction in the number of motifs considered and further simplifications (“Catchitt: a streamlined open-source implementation” section), Catchitt still yields competitive AUC-PR values. Ranking the Catchitt results within the original challenge results, we find that performance achieved by Catchitt scores is only two ranks lower than the challenge implementation using DNase-seq-based and motif-based features. As before, we find a substantial improvement of prediction performance due to the iterative training procedure.

## Discussion

Predicting in vivo binding sites of a TF of interest in silico is still one of the central challenges in regulatory genomics. A variety of tools and approaches for this purpose have been created over the last years and, among these, the approach presented here is not exceptional in many of its aspects. Specifically, it works on hand-crafted features derived from genomic and experimental data, it considers TF binding motifs and chromatin accessibility as its major sources of information, and it uses supervised learning related to logistic regression. Yet, this approach gained the best performance in the ENCODE-DREAM challenge. Notably, the second approach gaining the first rank in the challenge is based on a similar rationale and uses a set of DNase-seq-based and motif-based features in a logistic regression, where parameters are learned iteratively from systematically chosen sub-sets of the training cell types and chromosomes (cf. https://www.synapse.org/#!Synapse:syn7104742/wiki/407367).

In this paper, we focus on the impact of further, novel aspects of the proposed approach on prediction performance.

With regard to the features considered, we find that motif-based and DNase-seq-based features are pivotal for yielding a reasonable prediction performance for most TFs, while other sequence-based, annotation-based, or RNA-seq-based features have only marginal influence on the prediction result. In the case of RNA-seq-based features, however, more sophisticated features than those employed in our approach might have a positive influence on prediction accuracy. In addition, DNA shape might also be informative about true TF binding sites, although in silico shape predictions provided in ENCODE-DREAM are determined based on k-mers, and their influence might also be captured by higher-order Markov models or Slim/LSlim models [29] employed in the approach presented here.

Previous studies have shown that additional features like sequence conservation [22, 25], histone marks [13, 15, 19], or ChIP-seq data of co-factors [22] might also help to predict in vivo TF binding. However, these were not allowed to be used in the ENCODE-DREAM challenge and further experimental assays were unavailable for the training cell types. Hence, we decided to also exclude such features from the studies presented in this paper.

Two aspects of the presented approach, namely the iterative training procedure and aggregation of predictions across training cell types, contribute substantially to the final prediction performance. Both ideas might also be of relevance in related fields. Specifically, the iterative training procedure provides a general schema applicable to imbalanced classification problems, especially when these require sampling of negative examples. In an abstract sense, the aggregation across training cell types corresponds to favoring model averaging over model selection if good selection criteria are hard to find or might yield highly varying results.

Despite its state-of-the-art performance proven in the ENCODE-DREAM challenge, the approach presented here has important limitations. First, the large number of motifs (including those from de novo motif discovery) and DNase-seq-based features leads to high demands with regard to disk space but also runtime (cf. Additional file 1: Table S14), which are likely beyond reach for wet-lab biologists. Disk requirements could be reduced by computing features from (smaller) raw files on demand. However, this would in turn increase running time considerably. Hence, we chose to implement a simplified version of the approach presented here in an open-source software available at http://jstacs.de/index.php/Catchitt, which only uses a combination of chromatin accessibility features and motif-based features. In preliminary benchmarks (Additional file 1: Table S13), this implementation still achieved competitive performance.

Second, the approach proposed here, like any of the other supervised approaches [14–16, 21, 22, 24–26, 28], requires labeled training data for at least one cell type and the TF of interest to make predictions for this TF in another cell type. While the latter limitation is partly overcome by unsupervised approaches [13, 18–20, 23], this typically comes at the cost of reduced prediction accuracy [21, 25].

We also provide a large collection of 682 predicted peak files for 31 TFs using 22 DNase-seq data sets for primary cell types and tissues. Benchmarks based on the limited number of available ChIP-seq data indicate that prediction performance on these cell types is comparable to that achieved in the ENCODE-DREAM challenge, where absolute values of AUC-PR measuring prediction accuracy vary greatly between different TFs. For the wide majority of these combinations of TF and cell type, no experimental data about cell type-specific TF binding is available so far, which renders these predictions a valuable resource for questions related to regulatory genomics in these primary cell types and tissues. Preliminary studies raise our confidence that the predicted peak files may indeed help to solve biological questions related to these cell types and TFs.

## Methods

### Data

We use the following types of input data sets as provided by the challenge organizers (https://www.synapse.org/#!Synapse:syn6131484/wiki/402033):

- The raw sequence of the human genome (hg19) and gene annotations according to the gencode v19 annotation (http://www.gencodegenes.org/releases/19.html) [42]
- Cell type-specific DNase-seq “fold-enrichment coverage” tracks, which represent DNase-seq signal relative to a pseudo control, smoothed in a 150-bp window
- Cell type-specific DNase-seq peak files in “conservative” (IDR threshold of 10% in pseudo replicates) and “relaxed” (no IDR threshold) flavors
- Cell type-specific TPM values from RNA-seq experiments in two bio-replicates for all gencode v19 genes as estimated by RSEM [43]
- Cell type-specific and TF-specific ChIP-seq peak files in “conservative” (IDR threshold of 10% in pseudo replicates) and “relaxed” (no IDR threshold) flavors
- Cell type-specific and TF-specific label files classifying genome-wide 200-bp regions every 50 bp into B = “bound,” A = “ambiguous,” and U = “unbound” according to the respective conservative and relaxed ChIP-seq peak files; an overview of the combinations of TF and cell type in the training data, the leaderboard data, and the test data used for evaluation in the final challenge round is given in Additional file 1: Figure S1

In addition, we download sequence motifs represented as PWMs from the following collections:

- TF-specific motifs from the databases HOCOMOCO [44] and DBcorrDB [45]
- Motifs related to epigenetic markers from the epigram pipeline [46]

Details about the motifs considered are given in the “Features” section and Additional file 1: Text S2.

For predicting cell type-specific binding of TFs in additional cell types beyond those considered in the challenge, we download DNase-seq data (FastQ format) from the ENCODE project (http://www.encodeproject.org). Specifically, we select all DNase-seq experiments that (i) are flagged as “released,” (ii) have FastQ files available, (iii) are not from immortalized cell lines, (iv) have no entry in one of the “Audit error” categories, and (v) are not in the “insufficient replicate concordance” category of “Audit not compliant.” A list of the corresponding experiments is obtained from the ENCODE project, and experiments are filtered for the existence of at least two replicates, yielding 23 experiments in total. One of these experiments had to be excluded later, because a different DNase protocol with much shorter reads had been used. For the remaining 22 experiments (Additional file 1: Table S5), all FastQ files are downloaded from ENCODE and processed using ATAC-Seq/DNase-Seq Pipeline (https://github.com/kundajelab/atac_dnase_pipelines, latest git commit: c1d07d38a02af2f0319a69707eee047ab6112ecc (Tue Mar 21 20:31:25 2017)). The data sets are analyzed using the following parameters: -species hg19 -type dnase-seq -subsample 50M -se. For further analyses, the relaxed (./out/peak/idr/pseudo_reps/rep1/*.filt.narrowPeak.gz) and conservative peaks (./out/peak/macs2/overlap/*pval0.1*.filt.narrowPeak.gz) as well as the DNase coverage (./out/signal/macs2/rep1/*.fc.signal.bigwig) are used.

In addition, we download ChIP-seq peak files (Additional file 1: Table S6) matching these cell types and one of the TFs considered. Based on the “relaxed” (i.e., “optimal idr thresholded peaks”) and “conservative” (i.e., “conservative idr thresholded peaks”) peak files, we derive labels for 200-bp windows every 50 bp as proposed for the challenge. Specifically, we label each 200-bp region overlapping a conservative peak by at least 100 bp as “bound.” Of the remaining regions, all regions that overlap a relaxed peak by at least 1 bp are labeled “ambiguous,” while all other regions are labeled “unbound.” For a subset of TFs, no conservative peaks are available due to the lack of replicates. In such cases, we also use the relaxed peaks to assign “bound” labels.

### Binning the genome

**Fig. 5:**
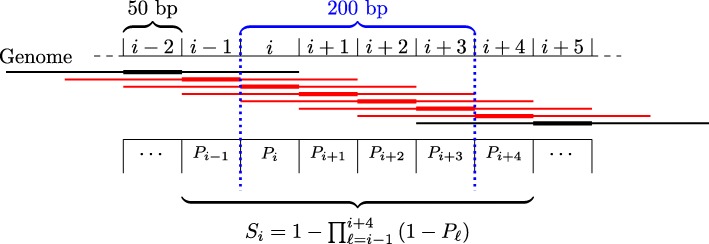
Schema for computing probabilities for regions overlapping with predicted peaks. We consider 200-bp regions and five bins in this example. Center bins are indicated by thick lines. Putative peaks are annotated with the probability *P*_*i*_ of being a true peak. All peaks marked in red overlap the region of interest (dotted blue lines) by at least 100 bp and are considered for the prediction. The prediction *S*_*i*_ for the 200-bp region is then computed as the probability that this region overlaps with at least one of the peaks

As the final prediction is requested for overlapping 200-bp regions with an offset of 50 bp, we decide to compute features with a matching resolution of 50 bp. To this end, the genome is divided into non-overlapping bins of 50 bp. Features are then either computed directly with that resolution (where possible, e.g., distance to the closest TSS) or first computed with base-pair resolution and afterwards summarized as aggregate values (minimum, maximum, median, or similar statistics) for each 50-bp bin. An odd number of several, adjacent bins, i.e., the respective feature values (see below), is then considered as input of the classifier composed of statistical models for the training process as well as for making predictions. Conceptually, the classifier uses the information from those bins to compute a posteriori probabilities *P*_*i*_ that center bin *i* (i.e., the central bin of those adjacent bins considered, cf. Fig. 5) contains a peak summit. The number of adjacent bins considered is determined from the median across cell types of the median peak widths of a given TF in the individual training cell types.

### Features

The set of features considered may be roughly classified by the source of information: DNase-seq data, motif profiles, raw sequence, RNA-seq data. Here, we give a brief overview of these features, while we provide a complete list of definitions of all features in Additional file 1: Text S2.

The most informative features with regard to the challenge task are likely motif-based and chromatin accessibility-based features. For obtaining a broad set of binding motifs for each TF at hand, we combine motifs from databases with motifs obtained by de novo motif discovery from the challenge data. We retrieve PWM models of the TF at hand from the databases HOCOMOCO [44] and DBcorrDB [45]. We perform de novo motif discovery with Dimont [47] learning PWM and LSlim(3) models [29] on the “conservative” and “relaxed” ChIP-seq peak files, and also based on the peak files obtained from DNase-seq experiments. In addition, we obtain motifs from the epigram pipeline [46], which are related to DNA methylation and histone marks of active promoters and enhancers. For a specific combination of cell type and TF, we also consider motifs of a set of “peer” motifs, which are determined from the literature (Factorbook, [48]) and by comparing the overlaps between the respective peak lists.

All of these motifs are then used in a sliding window approach to obtain base-pair resolution score profiles, which are summarized by aggregate statistics representing the binding affinity to the strongest binding site (i.e., the maximum log-probability in a bin according to the motif model) as well as general affinity to broader regions (i.e, the logarithm of the average probability in a bin). The set of motifs may comprise models of general binding affinity of the TF at hand but may also capture cell type-specific differences in the binding regions, which could be caused by interaction with other TFs including competition for similar binding sites.

DNase-seq-based features are computed from the “fold-enrichment coverage” tracks and DNase-seq peak files provided with the challenge data. These features quantify short and long range chromatin accessibility, stability of the DNase signal in the region of interest and across different cell types, and overlaps with DNase-seq peak regions.

The set of sequence-based features comprises the raw sequence (i.e., in 1-bp resolution) around the center bin and several measures computed from this sequence, for instance G/C-content, the frequency of CG di-nucleotides, or the length of homo-polymer tracts. Based on the gencode v19 genome annotation, we additionally define features based on overlapping annotation elements like CDS, UTRs, or TSS annotations and based on the distance to the closest TSS annotation in either strand orientation. All of these features are neither cell type-specific nor TF-specific. However, they may represent general features of genomic regions bound by TFs (like CpG islands, GC-rich promoters, or preference for non-coding regions), which might be helpful to rule out false positive predictions based on TF-specific features like motif scores. In addition, the model parameters referring to those features may be adapted in a TF-specific and cell type-specific manner, which may yield auxiliary information for cell type-specific prediction of TF binding as well.

Finally, RNA-seq data are represented by the TPM value of the gene closest to the bin of interest as well as measures of stability within biological replicates and across different cell types.

DNase-seq and RNA-seq-based features are cell type-specific but not TF-specific by design. However, model parameters may adapt to situations where one TF preferentially binds to open chromatin, whereas another TF may also bind in nucleosomal regions.

Feature values are computed using a combination of Perl scripts and Java classes implemented using the Java library Jstacs [49]. Genome-wide feature values with bin-level resolution are pre-computed and stored in a sparse, compressed text format.

### Model and basic learning principle

We model the joint distribution of these different features by a simple product of independent densities or discrete distributions (Additional file 1: Text S3). Specifically, we model numeric features (e.g., DNase-based statistics, motif scores, RNA-seq-based features) by Gaussian densities, discrete, annotation-based features by independent binomial distributions, and raw sequence by a homogeneous Markov model of order 3. All distributions are in the exponential family and parameterized using their natural parameterization [50, 51], which allows for unconstrained numerical optimization.

As learning principle, we use a weighted variant [52] of the discriminative maximum conditional likelihood principle ([53], Additional file 1: Text S3), which is closely related to logistic regression but allows for making explicit assumptions about the distribution of the underlying data.

### Prediction schema

In the challenge, final predictions have been requested for 200-bp windows shifted by 50 bp along the genome, while the proposed classifier predicts a posteriori probabilities that the current center bin contains a peak summit. To yield the predictions requested, we use these original prediction values (cf. “Binning the genome” section) to compute the probability that the 200-bp window overlaps at least one predicted peak by at least 100 bp (Fig. 5). Assume that we already computed the a posterior probabilities *P*_*i*_ that center bin *i* contains the summit of a ChIP-seq peak according to the trained model. Further assume that for the current TF, a peak typically spans five bins in total, which corresponds to the center bin and two bins before and two bins after the center bin in our model (cf. regions marked by lines in Fig. 5). Putative peaks overlapping the current 200-bp window starting at bin *i* are those with center bins at *i*−1 to *i*+4. Hence, the probability *S*_*i*_ that this window overlaps a peak may be computed as the complementary probability of the event that this window overlaps no predicted peaks, which in turn is just the product of the complementary a posteriori probabilities *P*_*ℓ*_ of these bins.

### Initial training data

For training the model parameters by the discriminative maximum condition likelihood principle, we need labeled input data comprising a set of positive (bound) regions and a set of negative (unbound) regions. In general, a training region is represented by a vector of all feature values described in the “Features” section in an odd number of consecutive bins (see the “Binning the genome” section). In case of positive regions, these are centered at the bin containing the peak summit. We include all such regions around the peak summits of the “conservative peaks” for the current TF and cell type as positive regions.

Since we face a highly imbalanced classification problem with rather few ChIP-seq peaks compared with the large number of bins not covered by a peak, and since the inclusion of all such negative regions into the training set would lead to an inacceptable runtime, we decided to derive representative negative regions by three different sampling strategies. All sampling steps are performed stratified by chromosome.

First, we sample on each training chromosome 10 times as many negative regions (spanning an odd number of consecutive bins) as we find positive regions on that chromosome. Center bins are sampled uniformly over all bins not covered by a “relaxed” peak for the same cell type and TF.

Second, we over-sample negative regions with large DNase-seq median values similar to those of positive examples to yield a representative set of negative regions. This is especially important as these will be regions that are hard to classify using DNase-seq based features but are only lowly represented by the uniform sampling schema. The over-sampling is adjusted for by down-weighting the drawn negative examples to the corresponding frequency among all negative regions (see Additional file 1: Text S4).

Third, we sample negative regions from regions that are ChIP-seq positive for one of the other cell types (if more than one training cell type exists for that TF), but do not overlap a “relaxed peak” in the current cell type. These negative regions are weighted such that the sum of their weights matches the rate of such regions among all putative negative regions. This sampling schema is intended to foster learning cell type-specific properties as opposed to general properties of the binding regions of the current TF. In this case, we sample four times as many negative regions as we have positives.

**Fig. 6:**
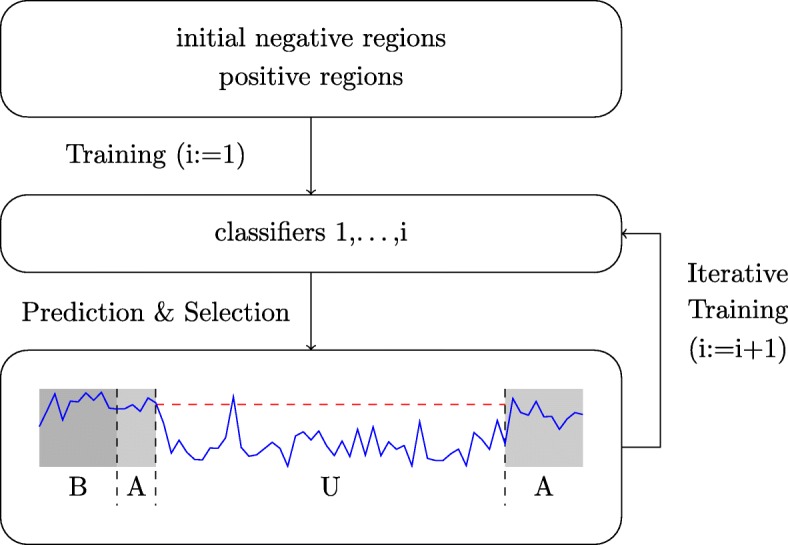
Iterative training procedure. Starting from an initial set of negative regions and the complete set of positive regions, a first classifier is trained and applied to the training data, and putative false positive (i.e., “unbound” regions with large prediction scores) are identified. In each of the subsequent iterations, such regions are added to the set of negative regions, which are in turn used for training refined classifiers. The result of this iterative training procedure is a set of five classifiers trained in five cycles of the iterative training procedure

Together, these three sampling schemas yield an initial set of negative regions, which serve as input of the discriminative maximum conditional likelihood principle in addition to the positive regions. However, in preliminary tests during the leaderboard round of the challenge, we observed that even this non-trivial sampling schema is not fully satisfactory. As testing (a large number of) other sampling schemas seemed futile, we designed an iterative training schema (Fig. 6) that is loosely related to boosting [54] and successively complements the initial set of negative training regions with further, informative examples.

### Iterative training

The iterative training procedure is illustrated in Fig. 6. Initially, we train a classifier on the negative regions obtained from the sampling schemas explained above and all positive regions. We then use this classifier to obtain a posteriori probabilities *P*_*i*_ for each bin *i* on training chromosomes. To limit the runtime required for this prediction step, we restrict the prediction to chromosomes chr10 to chr14. These probabilities are then used as input of the prediction schema (“Prediction schema” section) to yield predictions for the 200-bp regions labeled based on the ChIP-seq training peaks. Given these labels, we may distinguish prediction values of positive regions (label B = “bound”) and negative regions (label U = “unbound”), while regions labeled as A = “ambiguous” are ignored. To select additional negative regions that are likely false positive predictions, we first collect the prediction scores of all positive regions (labeled as B) and determine the corresponding 1% percentile. We then select from the negative regions (labeled as U) all those with a prediction score larger than this 1% percentile, which are subsequently added to the set of negative regions with a weight of 1 per region selected.

In the next iteration, we train a second classifier, again using all positive regions but with negative regions complemented by these additional negative regions. Prediction is then performed using both classifiers, where the predictions of the individual two classifiers (or all previously trained classifiers in subsequent iterations) are averaged per region. Again, regions labeled U with large prediction scores are identified and added to the set of negative regions, which then serve as input of the following iteration. After five rounds of training yielding five classifiers, the iterative training procedure is terminated.

### Final prediction

The iterative training procedure is executed for all *K* training cell types with ChIP-seq data for the TF of interest, which yields a total of 5·*K* classifiers. For the final prediction, the prediction schema (“Prediction schema” section) is applied to all chromosomes and each classifier. These predictions are finally averaged per 200-bp region to yield the final prediction result.

### Catchitt: a streamlined open-source implementation

Since the original challenge submission, we have re-implemented the basic approach with the aim of making it more accessible for both, users and developers. Specifically, our objectives were to implement a tool that (i) is consolidated into a single runnable JAR file to limit system requirements to a current Java installation only, (ii) has an extensible code base eliminating much of the experimental code of the challenge implementation, (iii) is applicable to data from individual cell types to reduce data interdependencies, and (iv) may be executed on a standard compute server in acceptable runtime.

To achieve these aims, some parts of the methods have been simplified and streamlined. First, we consider only the most important chromatin accessibility and motif-based features, which reduces runtime and memory consumption. Second, we implement an accelerated motif scanning module that computes whole-genome score profiles even for the complex LSlim models within a few hours. Third, we skip steps that jointly evaluate data and/or feature files from multiple cell types. Specifically, we skip quantile normalization of chromatin accessibility features (although normalization could be performed externally, still), and we omit the sampling step depending on ChIP-seq data for other cell types for determining initial negative regions. We call this implementation “Catchitt” comprising five modules for (i) computing chromatin accessiblity features from DNase-seq or ATAC-seq data, (ii) computing motif-based features, (iii) deriving labels from ChIP-seq peak lists, (iv) performing iterative training given feature files and labels, and (v) predicting binding probabilities for genomic regions.

### Implementation

The models and basic conditional likelihood training including numerical optimization are implemented by core classes of the Java library Jstacs [49]. The iterative training procedure and prediction schema have been implemented specifically for the challenge and have been further refined in the Catchitt implementation. Further details about the implementation are given in Additional file 1: Text S5.

### Deriving peak lists

For the additional primary cell types and tissues beyond those considered in the challenge, we further process final predictions to yield peak lists in narrowPeak format, which are smaller and easier to handle than the genome-wide probability tracks with 50-bp resolution. To this end, we join contiguous stretches of regions with predicted binding probability above a pre-defined threshold *t* into a common peak region. For each region, we record the maximum probability *p* and discard bordering regions with a probability below 0.8·*p*. The resulting regions are then annotated according to the narrowPeak format with a “peak summit” at the center of the region yielding *p*, a “score” of −100·*log*_10_(1−*p*), and a “signal value” equal to *p*. We generate “relaxed” peak predictions using *t*=0.6 and “conservative” peak prediction using *t*=0.8.

## Additional file

Additional file 1 Supplementary Tables and Figures. Figures S1 to S9. Tables S1 to S14. Text S1—Tools for predicting in vivo binding regions. Text S2—Features. Text S3—Model and learning principle. Text S4—Sampling of DNase-matched negative regions. Text S5—Implementation notes. (PDF 436 kb)
