# Supplementary material for: Accurate prediction of cell type-specific transcription factor binding
Source: Genome Biol. 2019 Jan 10;20:9. doi: 10.1186/s13059-018-1614-y (PMC6327544; doi:10.1186/s13059-018-1614-y)
Supplement: Supplementary file 1 — Supplementary Tables and Figures. Figures S1 to S9. Tables S1 to S14. Text S1—Tools for predicting in vivo binding regions. Text S2—Features. Text S3—Model and learning principle. Text S4—Sampling of DNase-matched negative regions. Text S5—Implementation notes. (PDF 436 kb) [file 13059_2018_1614_MOESM1_ESM.pdf]

Supplementary material
"Accurate prediction of cell type-specific transcription factor binding"

Jens Keilwagen, Stefan Posch, Jan Grau

Table S1: Previous approaches for predicting *in-vivo* transcription factor binding sites and their properties, listed in chronological order.

Approach	Motifs	Accessibility	additional Features	Learning	Model	specifics
CENTIPEDE [1]	PWMs (TRANS-FAC, Jaspar), de-novo k-mers	DNase-seq	histone modifications	unsupervised	hierarchical mixture model	first predict motifs then matched to DNase-seq profiles
[2]	PWMs (TRANS-FAC, Jaspar, UniProbe)	DNase-seq		supervised	Sparse logistic regression	predict gene regulation
[3]	k-mer based	DNase-seq	histone modifications	supervised	SVMs	cell type-specific binding motifs
Millipede [4]	PWMs (TRANS-FAC, Jaspar)	DNase-seq		supervised	logistic regression	same motifs as CENTIPEDE, binned DNase-seq cuts
Wellington [5]	PWMs (Homer)	DNase-seq		-	based on statistical tests	strand specific cut profiles
PIQ [6]	PWMs (TRANS-FAC, Jaspar, UniProbe)	DNase-seq		unsupervised	probabilistic model	first predict motifs then matched to DNase-seq profiles, high resolution
[7]	PWMs (TRANS-FAC, Jaspar, UniProbe)	DNase-seq	histone modifications	unsupervised	Hidden Markov model	active footprints annotated with motifs
msCentipede [8]	PWMs (from SE-LEX)	DNase-seq, ATAC-seq		unsupervised	hierarchical multi-scale model	explicitly models heterogeneities in cut profiles
BinDNase [9]	PWMs (Factor-book)	DNase-seq		supervised	logistic regression	high resolution, DNase-seq signal TF-specific
[10]	PWMs (Jaspar)	DNase-seq	*in-silico* nucleosome occupancy, structural features, conservation, ChIP-seq of co-factors	supervised	SVMs	also regression
Romulus [11]	motif matches (Homer)	DNase-seq	(histone modifications, conservation)	unsupervised (EM)	probabilistic model	motif matches used as prior information
FactorNet [12]	convolutional neural network	DNase-seq	mappability, annotations, CpG islands, expression	supervised (Deep learning)	convolutional-recurrent neural network	motif discovery part of deep learning
[13]	PWMs (TRANS-FAC)	DNase-seq (ATAC-seq)	conservation, distance to TSS	supervised	Random forests	model based on motif and DNase may be transferred across cell types and TFs
TFImpute [14]	convolutional neural network	(DNase-seq: negative training regions)		supervised (Deep learning), multi-task learning	deep neural network	complete matrix of cell type-TF combinations
TEPIC [15]	PWMs (Jaspar, UniProbe)	DNase-seq	(histone modifications)	-	TRAP scores with exponential distance prior	predict gene expression using elastic net
Mocap [16]	PWMs (ENCODE, CisBP)	DNase-seq	GC/CpG-content, mappability, distance to TSS, conservation	supervised	sparse logistic regression	three-stage model, ensemble classifier

Table S2: Final ranking of the ENCODE-DREAM challenge, reproduced from `https://www.synapse.org/#!Synapse:syn6131484/wiki/405275`. Briefly, final ranks are obtained by i) measuring performance in 10 bootstrap samples for each TF/cell type, ii) computing a normalized rank per bootstrap sample across all performance measures yielding 10 ranks per TF/cell type, iii) obtaining the total score per bootstrap sample as the average relative rank across all TFs/cell types in that bootstrap sample. The final total rank is the 90th percentile of average relative ranks across bootstrap samples per team. In addition, the lower bound, mean, and upper bound of the average relative ranks across bootstrap samples are listed.

Team name	rank	Lower bound	Mean	Upperbound
Yuanfang Guan	1.00	0.15	0.16	0.17
J-Team	1.00	0.16	0.16	0.17
dxquang	3.00	0.22	0.23	0.24
adbc	4.00	0.31	0.32	0.33
autosome.ru	5.00	0.32	0.33	0.34

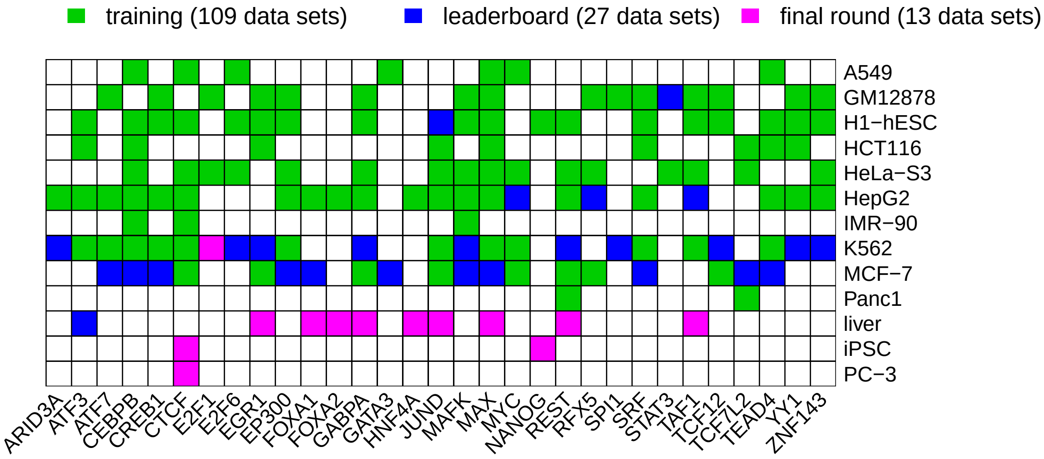

Fig. S1: Overview of the combinations of cell type and TF in the ENCODE-DREAM training, leaderboard, and final round sets.

Table S3: Ranking per TF/cell type in the ENCODE-DREAM challenge, reproduced from `https://www.synapse.org/#!Synapse:syn6131484/wiki/405275`, including the approach proposed in this paper (J-Team). Ranks per TF/cell type are obtained by i) measuring performance in 10 bootstrap samples for each TF/cell type, ii) computing a normalized rank per bootstrap sample across all performance measures yielding 10 ranks per TF/cell type. The final rank of each team per TF/cell type is the 90th percentile of the bootstrap ranks. Performance measures listed in the table are those obtained for the first bootstrap sample. Hence, the AUC-PR values reported for the approach proposed in this paper (J-Team) may slightly deviate from the AUC-PR values reported on the complete test data sets.

Rank	Team name	AUC-ROC	AUC-PR	Recall@50% FDR	Recall@10% FDR
CTCF PC-3					
1	dxquang	0.986217330227	0.782687521556	0.794840790004	0.613365022446
2	J-Team	0.975077470338	0.753448891593	0.763318461688	0.584330567485
3	davidaknowles	0.932199241358	0.69657574109	0.763846613574	0.396058319087
3	yuanfang.guan	0.963107525421	0.607931868275	0.596853326662	0.391499534392
5	adbc	0.951471924802	0.550438500262	0.523759885474	0.331915662483
CTCF IPSC					
1	dxquang	0.996617775166	0.860776696902	0.914165873908	0.684273112514
2	davidaknowles	0.995968442285	0.832481209884	0.881129464672	0.63698867076
3	J-Team	0.994996088948	0.81649114036	0.856525123238	0.595260745481
4	autosome.ru	0.992669982956	0.780153493659	0.80621378535	0.556127302603
4.5	adbc	0.993205095789	0.777274654504	0.809413646977	0.536928132837
E2F1 K562					
1	J-Team	0.992979168355	0.427395726529	0.37462406015	0.00507518796992
2	Ramil Nurtdinov	0.960490758995	0.365892167914	0.342763157895	0.0113721804511
3	adbc	0.99226015265	0.35103497284	0.288063909774	0.000281954887218
3	yuanfang.guan	0.995770339176	0.352142995523	0.232612781955	0.0
5	autosome.ru	0.991932293435	0.353042157779	0.188909774436	0.000281954887218
EGR1 liver					
1	yuanfang.guan	0.994078143398	0.428921587617	0.420699846941	0.000633345648388
1	J-Team	0.991594481586	0.398903018922	0.296880772682	0.0335673193645
3	adbc	0.991644480391	0.327827982558	0.179659048926	0.00902517548952
3	autosome.ru	0.987949560195	0.364234620215	0.238824088246	0.0229587797541
5	dxquang	0.985555420923	0.317247143546	0.216445875336	0.0286061117855
FOXA1 liver					
1	dxquang	0.986197386068	0.492221458375	0.488928008895	0.109978689892
1.5	J-Team	0.977418496248	0.488358001587	0.520383581951	0.110534605763
3	yuanfang.guan	0.970606845661	0.376415921821	0.352774946725	0.0358565737052
4	adbc	0.960870162105	0.337086720813	0.311312887983	0.0406281849347
5	autosome.ru	0.949911341567	0.309891024981	0.282358936348	0.0412767534513
FOXA2 liver					
1	yuanfang.guan	0.980036667035	0.460677694553	0.474909628017	0.0418626345862
2	J-Team	0.971150659879	0.38702712923	0.350021378318	0.102499319781
3	adbc	0.967708826097	0.340741341767	0.287480079294	0.0339720915769
4	Ramil Nurtdinov	0.93620672598	0.363007956194	0.33711664788	0.00754071597932
5	autosome.ru	0.959178221089	0.314581875275	0.257433824387	0.0171803941385
GABPA liver					
1	yuanfang.guan	0.991145376858	0.470382944432	0.391867905057	0.126109391125
2	dxquang	0.986077155658	0.441611731485	0.355005159959	0.145758513932
2.5	J-Team	0.982405992145	0.422831231929	0.360743034056	0.161981424149
4	autosome.ru	0.98336758817	0.443663093608	0.369742002064	0.0894117647059
5	adbc	0.980361549368	0.364394213755	0.281898864809	0.0951083591331
HNF4A liver					
1	dxquang	0.978494318401	0.618836893234	0.646708673306	0.24835215889
2	J-Team	0.971615180489	0.595929549214	0.610485205726	0.270928308023
3	yuanfang.guan	0.966758766328	0.585605500165	0.591219257237	0.293635122971
4	adbc	0.961352809308	0.529986835084	0.558625395627	0.158294375563
5	davidaknowles	0.966070765102	0.509607901615	0.539344928715	0.0959087081506
JUND liver					
1	yuanfang.guan	0.988833629534	0.59872470682	0.662027048361	0.112271746566
2	J-Team	0.978403674237	0.422307544136	0.385781192443	0.0720149450087
2.5	autosome.ru	0.977042232964	0.447356000712	0.423722570121	0.0481766036942
4	adbc	0.978748188296	0.367579456428	0.272720096827	0.0355996421618
5	HINT	0.9561941256	0.367828291983	0.284113034784	0.0543072146503
MAX liver					
1	yuanfang.guan	0.991746814903	0.535399657046	0.588478989939	0.0153876504241
2	HINT	0.95997584562	0.440231369964	0.481628526337	0.00969126060367
2	autosome.ru	0.982767662617	0.473930602296	0.505942986782	0.00724995068061
4	J-Team	0.985255809252	0.447635741394	0.458399092523	0.00342769777076
4	davidaknowles	0.988468222643	0.429393844769	0.414554152693	0.000493193923851
NANOG IPSC					
1	dxquang	0.988513719636	0.353926718784	0.311808986121	0.00317572335921
2	yuanfang.guan	0.989553792922	0.308691209204	0.244648318043	0.0399905904493
3	J-Team	0.987454494921	0.315239838124	0.266055045872	0.0278757939308
4	autosome.ru	0.983177611442	0.195105149801	0.0782168901435	0.0159962361797
5	HINT	0.977119267757	0.209531929706	0.11714890614	0.0
REST liver					
1	dxquang	0.980045420537	0.412170318171	0.406472168723	0.0281047150296
2	HINT	0.936732703562	0.411905089056	0.406686164016	0.0308628765723
3	yuanfang.guan	0.97069508862	0.39782135426	0.350880947286	0.00366169722044
4	J-Team	0.96290735932	0.264076103071	0.0605606676653	0.0317901895047
4	NittanyLions	0.81063427363	0.401257426838	0.499369902751	0.0
TAF1 liver					
1	yuanfang.guan	0.9917037538	0.437342094864	0.407124543124	0.000272761987889
2	J-Team	0.990046815991	0.412563658303	0.382521411816	0.0019638863128
3	adbc	0.991108118397	0.401538933991	0.34166166603	0.000163657192734
3	dxquang	0.989179476303	0.428313969763	0.403851399269	0.0
5	HINT	0.963369400393	0.28588792284	0.189242267198	0.00300038186678

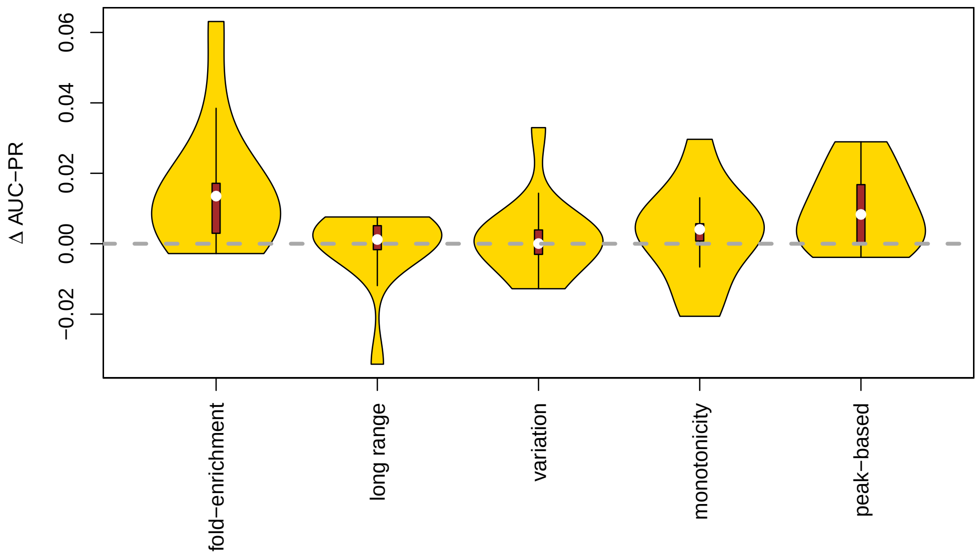

Fig. S2: Assessment of the importance of different groups of DNase features by excluding the respective group of features from the training data. The measure ΔAUC-PR is computed as the difference of AUC-PR with all features included and AUC-PR with one group of features left out. Hence, positive values indicates that the inclusion of a specific group of features leads to an improvement in prediction performance. In general, we find that excluding any of the groups has only minor impact on performance. This is likely due to wide redundancies and correlations among the different DNase features, which may help to compensate for the loss of a specific group.

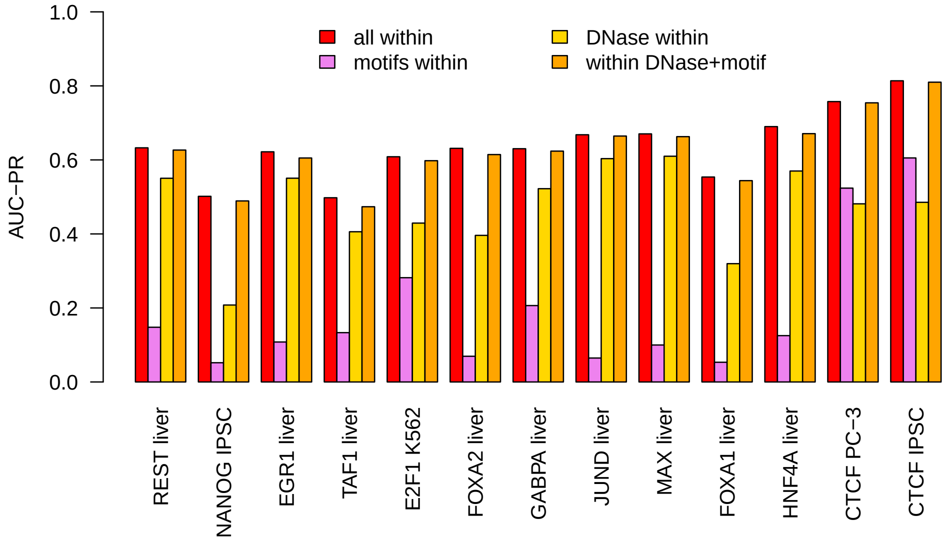

Fig. S3: Within cell type performance. For each of the 13 combinations of TF and cell type within the test data, we compute the prediction performance (AUC-PR) on the held-out chromosomes of classifiers i) using all features considered, ii) using only motif-based features, iii) using only DNase-seq-based features, and iv) using only motif-based and DNase-seq-based features. The training data comprises the training chromosomes of the same (test) cell type, while predictions are made for the held-out test chromosomes of that cell type.

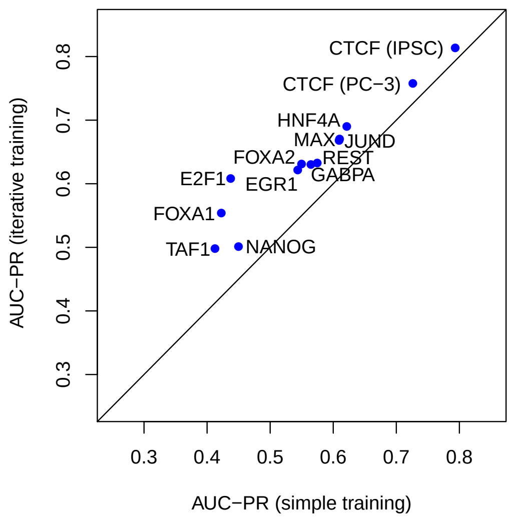

Fig. S4: Relevance of the iterative training procedure for within cell type predictions. For each of the 13 test data sets, we compare the performance (AUC-PR) achieved by the (set of) classifier(s) trained on the initial negative regions (abscissa) with the performance achieved by averaging over all classifiers from the iterative training procedure (ordinate). The training data comprises the training chromosomes of the same (test) cell type, while predictions are made for the held-out test chromosomes of that cell type.

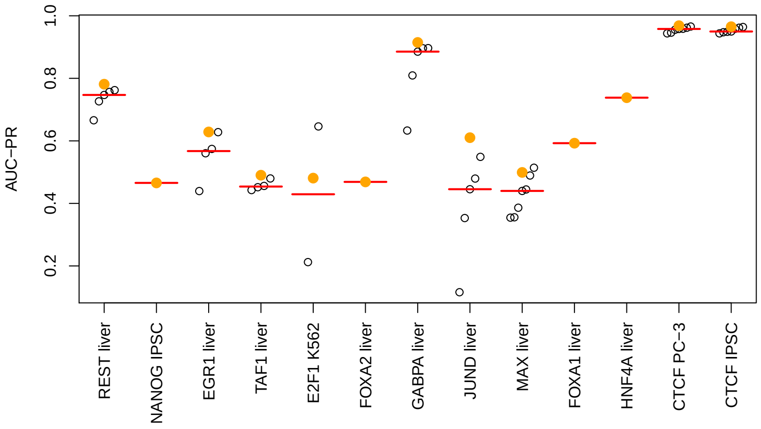

A

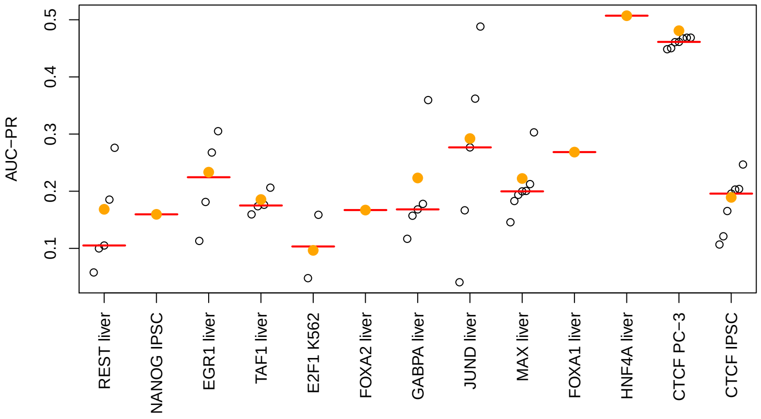

B

Fig. S5: Assessment of prediction performance separately on regions conserved (A) and variable (B) among cell types. We consider a bound region conserved if it is also labeled as “bound” in at least 3 of 4 training cell types, and we consider a bound region as variable if this region is also labeled as “bound” in at most 1 of 4 training cell types. For each of the 13 test data sets, we compare the performance (AUC-PR) of the individual classifiers trained on single cell types (open circles) to that of the ensemble classifier averaging over all classifiers trained on all training cell types (filled, orange circles). As a reference, we also plot the median of the individual classifiers as a red bar.

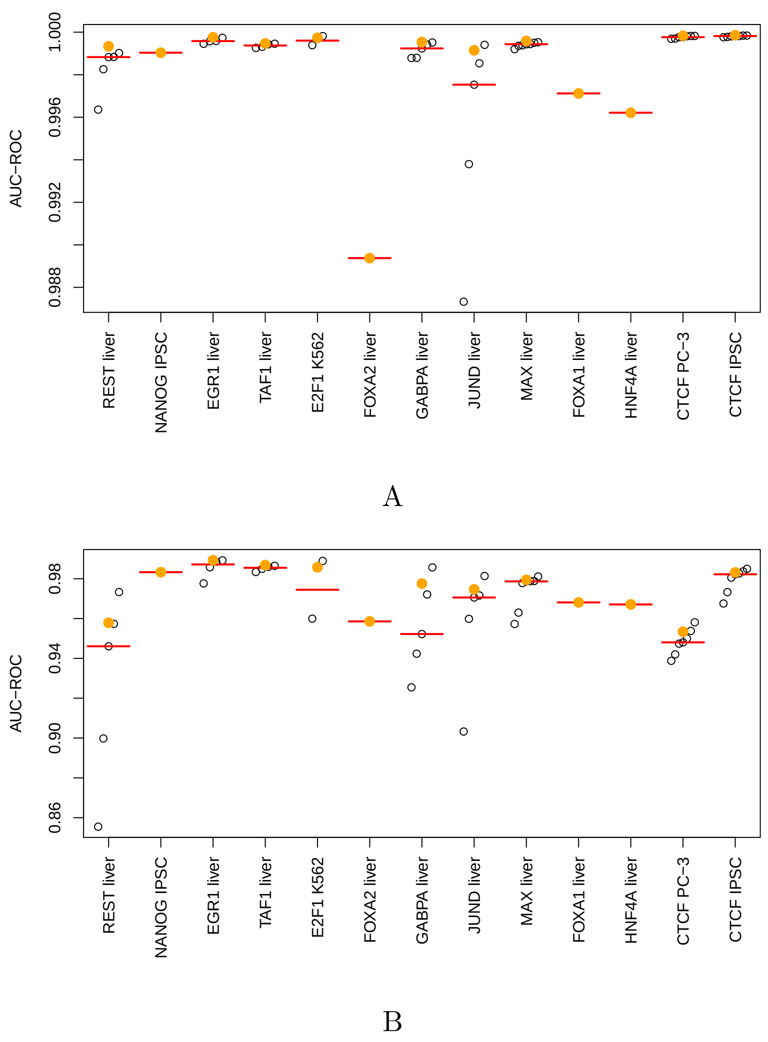

Fig. S6: Assessment of prediction performance separately on regions conserved (A) and variable (B) among cell types. We consider a bound region conserved if it is also labeled as "bound" in at least 3 of 4 training cell types, and we consider a bound region as variable if this region is also labeled as "bound" in at most 1 of 4 training cell types. For each of the 13 test data sets, we compare the performance (AUC-ROC) of the individual classifiers trained on single cell types (open circles) to that of the ensemble classifier averaging over all classifiers trained on all training cell types (filled, orange circles). As a reference, we also plot the median of the individual classifiers as a red bar.

Table S4: Performance (AUC-PR) on the test cell types using different sets of features. Columns “all features”, “motif-based”, “DNase-seq-based”, and “motif & DNase-seq-based” correspond to classifiers using only those feature sets, while columns with prefix “w/o” indicate that the given feature set has been excluded when training the classifiers (for details see main text, Figures 3 and 4).

TF	cell type	all features	motif-based	DNase-seq-based	motif & DNase-seq-based	w/o DNase	w/o motifs	w/o de-novo motifs	w/o Slim/LSlim motifs	w/o RNA-seq	w/o annotation	w/o sequence
CTCF	IPSC	0.807	0.5989	0.479	0.806	0.6028	0.576	0.763	0.778	0.807	0.807	0.807
CTCF	PC-3	0.747	0.5202	0.487	0.745	0.5307	0.572	0.707	0.721	0.747	0.747	0.746
E2F1	K562	0.388	0.2287	0.390	0.382	0.3017	0.326	0.366	0.382	0.384	0.390	0.385
EGR1	liver	0.377	0.0937	0.435	0.376	0.1242	0.354	0.366	0.375	0.375	0.377	0.378
FOXA1	liver	0.487	0.0538	0.259	0.482	0.0713	0.321	0.458	0.478	0.487	0.488	0.482
FOXA2	liver	0.392	0.0460	0.338	0.397	0.0642	0.358	0.443	0.426	0.396	0.392	0.391
GABPA	liver	0.410	0.1868	0.390	0.413	0.2289	0.391	0.412	0.409	0.409	0.411	0.414
HNF4A	liver	0.587	0.1110	0.430	0.577	0.1471	0.509	0.573	0.573	0.586	0.586	0.579
JUND	liver	0.420	0.0446	0.525	0.425	0.0588	0.499	0.438	0.435	0.418	0.419	0.427
MAX	liver	0.424	0.0654	0.485	0.426	0.0928	0.411	0.424	0.422	0.424	0.425	0.426
NANOG	IPSC	0.311	0.0226	0.181	0.319	0.0304	0.291	0.306	0.306	0.313	0.312	0.317
REST	liver	0.251	0.1033	0.180	0.250	0.1315	0.178	0.220	0.230	0.248	0.254	0.250
TAF1	liver	0.383	0.1133	0.360	0.366	0.1720	0.375	0.382	0.384	0.378	0.382	0.381

Table S5: Experiment IDs, tissue/cell type information, and biosample "Term ID" of the ENCODE DNase-seq data used in this study. The list of experiments was obtained from `https://www.encodeproject.org/report.tsv?type=Experiment&assay_title=DNase-seq&status=released&assembly=hg19&files.file_type=fastq&audit.NOT_COMPLIANT.category`. (accessed March 2, 2017)

Experiment ID	Donor ID	Tissue/Cell Type	Term ID
ENCSR000ENA	ENCDO223AAA	astrocyte of the hippocampus	CL:0002604
ENCSR000ENB	ENCDO224AAA	astrocyte of the spinal cord	CL:0002606
ENCSR000ENH	ENCDO095AAA	cardiac fibroblast	CL:0002548
ENCSR000ENJ	ENCDO330AAA	cardiac muscle cell	CL:0000746
ENCSR000ENN	ENCDO104AAA	epithelial cell of esophagus	CL:0002252
ENCSR000ENQ	ENCDO232AAA	foreskin fibroblast	CL:1001608
ENCSR000ENT	ENCDO100AAA	iris pigment epithelial	CL:0002565
ENCSR000EOE	ENCDO238AAA	lung microvascular endothelial	CL:2000016
ENCSR000ENZ	ENCDO241AAA	dermis blood vessel endothelial	CL:2000010
ENCSR000EOB	ENCDO243AAA	dermis microvascular lymphatic vessel endothelial	CL:2000041
ENCSR000EOQ	ENCDO000AAS	endothelial of umbilical vein	CL:0002618
ENCSR000EOR	ENCDO253AAA	fibroblast of villous mesenchyme	CL:0002558
ENCSR000EPP	ENCDO191CQJ	foreskin fibroblast	CL:1001608
ENCSR000EPR	ENCDO269AAA	fibroblast of lung	CL:0002553
ENCSR000EQC	ENCDO334AAA	T-helper 1 primary cell	CL:0000545
ENCSR000EMB	ENCDO442SWC	fibroblast of skin abdomen male adult (22 years)	CL:2000013
ENCSR000EMJ	ENCDO114AAA	B primary cell female adult (43 years)	CL:0000236
ENCSR621ENC	ENCDO539WIJ	retina tissue fetal (74 days)	UBERON:0000966
ENCSR474GZQ	ENCDO225GSN	retina tissue fetal (125 days)	UBERON:0000966
ENCSR503HIB	ENCDO240JUB	cerebellar cortex tissue male adult (84 years)	UBERON:0002129
ENCSR627NIF	ENCDO652XOU	lung tissue male fetal (58 days)	UBERON:0002048
ENCSR657DFR	ENCDO271OUW	thyroid gland tissue female adult (51 year)	UBERON:0002046

Table S6: ChIP-seq data sets available for the primary cell types and tissues. The last seven ChIP-seq data sets provide only "relaxed" peak lists.

TF	Experiment ID	File ID	Donor ID	Tissue/Cell Type	Type
CTCF	ENCSR000DSU	ENCFF312HCK	ENCDO224AAA	astrocyte of the spinal cord	relaxed
CTCF	ENCSR000DSU	ENCFF787GLH	ENCDO224AAA	astrocyte of the spinal cord	conservative
CTCF	ENCSR000DTI	ENCFF266GGD	ENCDO330AAA	cardiac muscle cell	relaxed
CTCF	ENCSR000DTI	ENCFF386NQE	ENCDO330AAA	cardiac muscle cell	conservative
CTCF	ENCSR000DTR	ENCFF528VFN	ENCDO104AAA	epithelial cell of esophagus	relaxed
CTCF	ENCSR000DTR	ENCFF373BXG	ENCDO104AAA	epithelial cell of esophagus	conservative
CTCF	ENCSR000DPM	ENCFF681OWQ	ENCDO001AAA	fibroblast of lung	relaxed
CTCF	ENCSR000DPM	ENCFF138PXI	ENCDO001AAA	fibroblast of lung	conservative
CTCF	ENCSR000DVQ	ENCFF738CXX	ENCDO253AAA	fibroblast of villous mesenchyme	relaxed
CTCF	ENCSR000DVQ	ENCFF199ZDU	ENCDO253AAA	fibroblast of villous mesenchyme	conservative
CTCF	ENCSR000DWQ	ENCFF337WIE	ENCDO191CQJ	foreskin fibroblast	relaxed
CTCF	ENCSR000DWQ	ENCFF275AVH	ENCDO191CQJ	foreskin fibroblast	conservative
CTCF	ENCSR000DLW	ENCFF002DBA	ENCDO000AAS	endothelial cell of umbilical vein	relaxed
CTCF	ENCSR000DWY	ENCFF002DDO	ENCDO269AAA	fibroblast of lung	relaxed
CTCF	ENCSR000DUH	ENCFF002DCY	ENCDO232AAA	foreskin fibroblast	relaxed
CTCF	ENCSR000DQI	ENCFF649IRT	ENCDO000AAG	foreskin fibroblast	relaxed
JUN	ENCSR000EFA	ENCFF002CVC	ENCDO000AAS	endothelial cell of umbilical vein	relaxed
MAX	ENCSR000EEZ	ENCFF002CVE	ENCDO000AAS	endothelial cell of umbilical vein	relaxed
MYC	ENCSR000DLU	ENCFF002DAZ	ENCDO000AAS	endothelial cell of umbilical vein	relaxed

Table S7: Prediction performance on primary cell types and tissues using labels derived from ChIP-seq data. Here, we include all performance measures considered in the ENCODE-DREAM challenge.
*: labels determined from only relaxed peaks.

TF	DNase ID	Experiment ID	Matching donor	AUC-ROC	AUC-PR	recall @ 10% FDR	recall @ 50% FDR
CTCF	ENCSR000ENB	ENCSR000DSU	yes	0.9953	0.7895	0.5603	0.8240
CTCF	ENCSR000ENJ	ENCSR000DTI	yes	0.9950	0.8197	0.6316	0.8486
CTCF	ENCSR000ENN	ENCSR000DTR	yes	0.9932	0.7788	0.5621	0.8098
CTCF	ENCSR000EPP	ENCSR000DWQ	yes	0.9939	0.7720	0.5517	0.7975
CTCF	ENCSR000EOR	ENCSR000DVQ	yes	0.9939	0.8048	0.6094	0.8319
CTCF	ENCSR000EPR	ENCSR000DPM	no	0.9913	0.7322	0.4834	0.7600
CTCF*	ENCSR000EOQ	ENCSR000DLW	yes	0.9962	0.7270	0.4030	0.7868
JUN*	ENCSR000EOQ	ENCSR000EFA	yes	0.9965	0.631	0.1644	0.6996
MAX*	ENCSR000EOQ	ENCSR000EEZ	yes	0.9967	0.4004	0.0255	0.3327
MYC*	ENCSR000EOQ	ENCSR000DLU	yes	0.9977	0.1989	0.000	0.0336

Table S8: ChIP-seq replicate peak files and corresponding IDR-filtered peak files for CTCF and different cell types. Experiment IDs match a subset of those listed in Table S6.

Replicate peaks	IDR peaks	Experiment ID	Biosamples	Donor
ENCFF729AWY,ENCFF892VYV	ENCFF312HCK	ENCSR000DSU	ENCBS302AAA,ENCBS995OME	ENCDO224AAA
ENCFF574BPV,ENCFF883SBZ	ENCFF266GGD	ENCSR000DTI	ENCBS887NMD,ENCBS465QGC	ENCDO330AAA
ENCFF015JHG,ENCFF114UPJ	ENCFF528VFN	ENCSR000DTR	ENCBS044KJV,ENCBS191CHS	ENCDO104AAA
ENCFF290QTC,ENCFF683BND	ENCFF337WIE	ENCSR000DWQ	ENCBS748WVA,ENCBS271PJP	ENCDO191CQJ
ENCFF493VQC,ENCFF777VSN	ENCFF649IRT	ENCSR000DQI	ENCBS488ZLU,ENCBS693PNC	ENCDO000AAG

Table S9: Jaccard coefficients between predicted (columns) and experimentally determined (rows) peak files for CTCF. Entries of matching tissues/cell types are marked in bold. In each row, we mark the largest value in green for matching cell types and in red for differing cell types. We mark matching donor with "(y)". Jaccard coefficients are computed using the `intersect` and `union` of the GenomicRanges R package. For each peak list, entries are sorted by score and limited to the minimum number of peaks across all peak lists. We find that many of the cell type-specific predictions for CTCF are more similar to the ChIP-seq peaks determined for "endothelial cells of umbilical vein" than to those of their cell type of origin according to the DNase-seq data. One reason might be that only for this experiment (ENCSR000DLW), peaks have not been called using the uniform ENCODE pipeline including SPP [17], but by another, "unknown" software. However, if we in turn ask for each experimentally determined peak list, which of the predicted peak lists is the most similar one, this picture becomes more encouraging. For 7 of the 8 cell types with matching donor between ChIP-seq and DNase-seq data, the most similar prediction is obtained for the true cell type, while in one case ("fibroblast of lung"), the most similar cell type is "foreskin fibroblast".

	astrocyte of the spinal cord (ENCSR000ENB)	cardiac muscle cell (ENCSR000ENJ)	endothelial cell of umbilical vein (ENCSR000EOQ)	epithelial cell of esophagus (ENCSR000ENN)	fibroblast of lung (ENCSR000EPR)	fibroblast of villous mesenchyme (ENCSR000EOR)	foreskin fibroblast (ENCSR000ENQ)	foreskin fibroblast (ENCSR000EPP)
astrocyte of the spinal cord (ENCSR000DSU)	**0.5852** (y)	0.5732	0.5465	0.5555	0.5675	0.5659	0.5725	0.5537
cardiac muscle cell (ENCSR000DTI)	0.5503	**0.5754** (y)	0.5309	0.5287	0.5408	0.5540	0.5517	0.5395
endothelial cell of umbilical vein (ENCSR000DLW)	0.6995	0.7049	**0.7090** (y)	0.6849	0.6730	0.6924	0.6888	0.6664
epithelial cell of esophagus (ENCSR000DTR)	0.5228	0.5230	0.4998	**0.5576** (y)	0.5123	0.5147	0.5214	0.5014
fibroblast of lung (ENCSR000DPM)	0.5361	0.5372	0.5119	0.5094	**0.5257**	0.5338	0.5383	0.5290
fibroblast of lung (ENCSR000DWY)	0.6451	0.6478	0.6197	0.6204	**0.6391** **(y)**	0.6497	0.6530	0.6501
fibroblast of villous mesenchyme (ENCSR000DVQ)	0.5818	0.5977	0.5629	0.5663	0.5737	**0.6084** (y)	0.5938	0.5758
foreskin fibroblast (ENCSR000DQI)	0.5752	0.5787	0.5558	0.5549	0.5723	0.5837	**0.5920**	**0.5850**
foreskin fibroblast (ENCSR000DUH)	0.6714	0.6755	0.6447	0.6509	0.6637	0.6787	**0.6934** (y)	**0.6770**
foreskin fibroblast (ENCSR000DWQ)	0.5400	0.5402	0.5141	0.5086	0.5301	0.5418	**0.5469**	**0.5680** (y)

Table S10: Jaccard coefficients between pairs of experimentally determined ChIP-seq peak files for CTCF. The last three rows/columns may be perceived as biological replicates of the same tissue but from different donors.

	astrocyte of the spinal cord (ENCSR000DSU)	cardiac muscle cell (ENCSR000DTI)	endothelial cell of umbilical vein (ENCSR000DLW)	epithelial cell of esophagus (ENCSR000DTR)	fibroblast of lung (ENCSR000DPM)	fibroblast of lung (ENCSR000DWY)	fibroblast of villous mesenchyme (ENCSR000DVQ)	foreskin fibroblast (ENCSR000DQI)	foreskin fibroblast (ENCSR000DUH)	foreskin fibroblast (ENCSR000DWQ)
astrocyte of the spinal cord (ENCSR000DSU)	1.0000	0.7009	0.5792	0.5976	0.6833	0.6176	0.6994	0.6826	0.6495	0.6271
cardiac muscle cell (ENCSR000DTI)	0.7009	1.0000	0.5691	0.5783	0.6783	0.6088	0.7129	0.6686	0.6258	0.6391
endothelial cell of umbilical vein (ENCSR000DLW)	0.5792	0.5691	1.0000	0.5387	0.5439	0.6619	0.5976	0.5835	0.6931	0.5511
epithelial cell of esophagus (ENCSR000DTR)	0.5976	0.5783	0.5387	1.0000	0.5765	0.5389	0.5936	0.5681	0.5633	0.5519
fibroblast of lung (ENCSR000DPM)	0.6833	0.6783	0.5439	0.5765	1.0000	0.6191	0.6925	0.6806	0.6331	0.6688
fibroblast of lung (ENCSR000DWY)	0.6176	0.6088	0.6619	0.5389	0.6191	1.0000	0.6479	0.6743	0.7875	0.6396
fibroblast of villous mesenchyme (ENCSR000DVQ)	0.6994	0.7129	0.5976	0.5936	0.6925	0.6479	1.0000	0.6870	0.6748	0.6548
foreskin fibroblast (ENCSR000DQI)	0.6826	0.6686	0.5835	0.5681	0.6806	0.6743	0.6870	1.0000	0.7200	0.6817
foreskin fibroblast (ENCSR000DUH)	0.6495	0.6258	0.6931	0.5633	0.6331	0.7875	0.6748	0.7200	1.0000	0.6580
foreskin fibroblast (ENCSR000DWQ)	0.6271	0.6391	0.5511	0.5519	0.6688	0.6396	0.6548	0.6817	0.6580	1.0000

Table S11: Jaccard coefficient between experimentally determined and predicted peak files. Jaccard coefficients are computed using the `intersect` and `union` of the GenomicRanges R package. For each TF, entries of the experimentally determined and predicted peak lists are sorted by score and limited to the minimum number of peaks in either of the two peak lists.

**TF**	**ChIP-seq**	**Predicted**	**Matching donor**	**Jaccard coefficient**
JUN	endothelial cell of umbilical vein (ENCSR000EFA)	endothelial cell of umbilical vein (ENCSR000EOQ)	yes	0.4500
MAX	endothelial cell of umbilical vein (ENCSR000EEZ)	endothelial cell of umbilical vein (ENCSR000EOQ)	yes	0.3634
MYC	endothelial cell of umbilical vein (ENCSR000DLU)	endothelial cell of umbilical vein (ENCSR000EOQ)	yes	0.2221

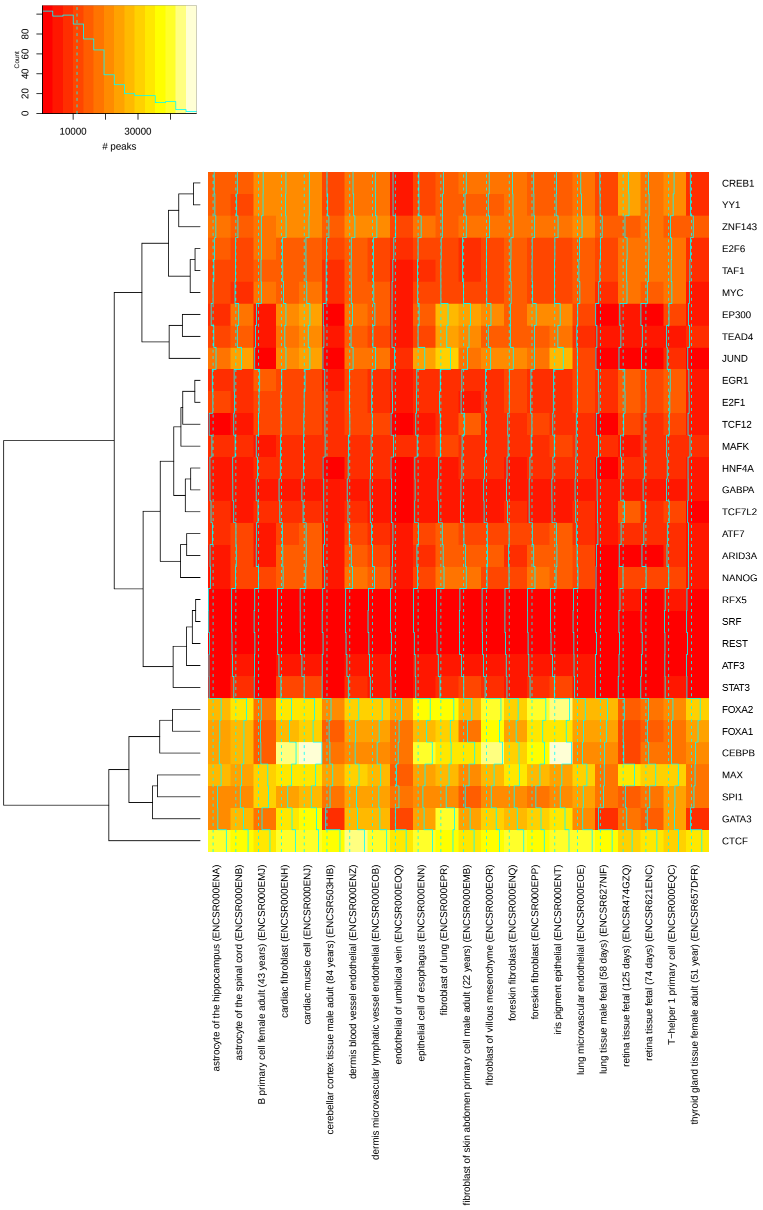

Fig. S7: Number of predicted peaks in "conservative" peak files for the studied TFs (rows) in the collection of primary cell types and tissues (columns). In each column of the heatmap, cyan trace lines in addition to colors indicate the corresponding values in each cell. In the color scale, the solid cyan line represents the histogram of values observed in the heatmap. Dashed lines indicate median values across all displayed numbers. Rows are clustered by the R `hclust` function using complete linkage.

Table S12: Jaccard coefficient between IDR-thresholded peaks and predicted peaks (cf.Table S9), between IDR-thresholded peaks and peaks called from the individual technical replicates, and between technical replicates.

Experiment ID	DNase prediction	IDR vs. Predictions	IDR vs. Rep1	IDR vs. Rep2	Rep1 vs. Rep2
ENCSR000DSU	ENCSR000ENB	0.5852	0.7440	0.7331	0.8444
ENCSR000DTI	ENCSR000ENJ	0.5754	0.7049	0.7239	0.8763
ENCSR000DTR	ENCSR000ENN	0.5576	0.7317	0.6965	0.8024
ENCSR000DWQ	ENCSR000ENQ	0.5469	0.7604	0.7539	0.8614
ENCSR000DWQ	ENCSR000EPP	0.5680	0.7604	0.7539	0.8614
ENCSR000DQI	ENCSR000ENQ	0.5920	0.8060	0.7908	0.8641
ENCSR000DQI	ENCSR000EPP	0.5850	0.8060	0.7908	0.8641

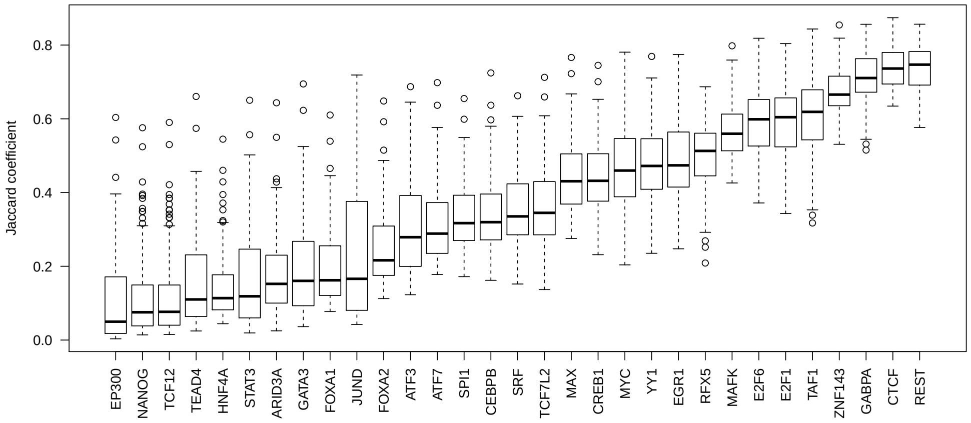

Fig. S8: Jaccard coefficients of the different TFs computed on the overlap of the peak files between all pairs of the 22 individual cell types.

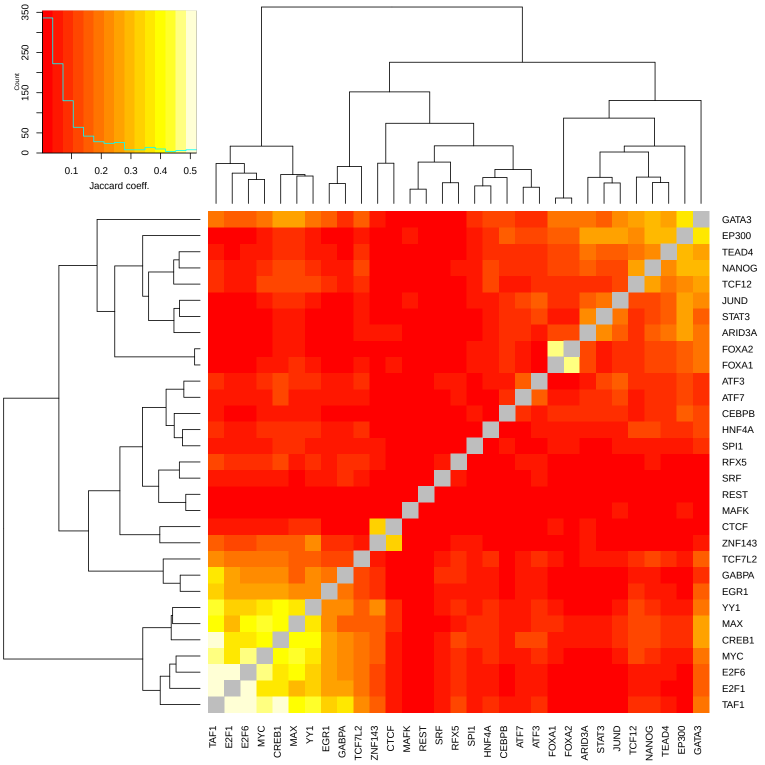

Fig. S9: Average Jaccard coefficients computed on the overlap of the peak files of pairs of TFs for matched cell types. In the color scale, the solid cyan line represents the histogram of values observed in the heatmap. Dashed lines indicate the value at the center bin of the color scale. Rows and columns are clustered by the R `hclust` function using complete linkage.

TF	cell type	DNase+motif			Catchitt			
		AUC-PR	Rank	#motifs	AUC-PR	Rank	#motifs	first iteration
CTCF	IPSC	0.810	4	177	0.776	6	13	0.760
NANOG	IPSC	0.489	2	127	0.404	4	13	0.366
FOXA1	liver	0.544	2	121	0.465	4	12	0.435
HNF4A	liver	0.671	4	123	0.615	6	10	0.597
TAF1	liver	0.473	4	160	0.412	6	12	0.400

Table S13: Benchmark of the simplified open source implementation (Catchitt) of the presented approach compared with the challenge implementation using only DNase-based and motif-based features for the within cell type case (cf. Fig. S3). For each of the TFs considered, we report AUC-PR achieved by the challenge implementation using only DNase-based and motif-based features ("DNase+motif"), the open source Catchitt implementation. For Catchitt, we additionally consider using only the classifier of the first iteration (in analogy to the comparison in Fig. S4). We also list the number of motifs utilized in the respective runs for a specific TFs. For the Catchitt runs, we deliberately limited the number of motifs considered to approximate a real-world application of the software. We finally report the ranks among the challenge participants according to the results available at `https://www.synapse.org/#!Synapse:syn6131484/wiki/412905`.

TF	Initial extraction	Training	Iter. extraction	Iter. prediction	Total	Catchitt
ARID3A	115	538	86	477	1216	
ATF2	118	640	89	612	1458	
ATF3	153	1338	113	648	2252	
ATF7	133	1573	97	665	2467	
CEBPB	163	3200	116	582	4062	
CREB1	132	1027	100	581	1840	
CTCF	157	2576	114	567	3414	1874
E2F1	108	356	80	503	1046	
E2F6	126	731	99	579	1536	
EGR1	125	705	95	530	1454	
EP300	156	1593	115	672	2536	
FOXA1	110	1078	83	400	1671	397
FOXA2	109	1851	78	442	2480	
GABPA	130	552	103	592	1377	
GATA3	106	597	82	457	1242	
HNF4A	103	553	82	401	1139	910
JUND	150	2153	112	652	3068	
MAFK	143	1408	110	552	2213	
MAX	160	2130	117	718	3124	
MYC	142	1016	109	581	1848	
NANOG	99	203	79	408	789	405
REST	152	1150	117	586	2006	
RFX5	136	854	107	610	1707	
SPI1	110	1000	83	400	1593	
SRF	151	993	117	647	1908	
STAT3	114	532	86	481	1213	
TAF1	140	948	108	624	1819	348
TCF12	119	771	92	523	1505	
TCF7L2	137	889	102	722	1850	
TEAD4	159	1650	115	716	2639	
YY1	149	1298	107	602	2157	
ZNF143	145	1685	102	545	2478	

Table S14: Runtime (in minutes) of the original challenge implementation using the full set of features. We separately list the average runtime per TF for i) the initial extraction of features values, ii) the training of parameters given the training data, summed over all iterations, iii) the extraction of further examples in the iterations, and iv) the prediction in the iterations. Finally, we list the total runtime of the complete iterative training, i.e., the sum over the previous columns. For comparison, we further list the total runtimes of the Catchitt runs (cf. Table S13) for five TFs. In addition to the iterative training, further runtime is required for generating features files from raw files. The extraction of features from DNase-seq data (bigwig format) takes approximately 30 min in both implementations. The implementation for scanning genomes with PWMs is slightly more efficient in Catchitt (20 min) compared with the original challenge implementation (30 min), which computes some additional features. Runtime improvement is especially pronounced for genome-wide scans using complex Slim models: while a genome-wide scan using a full Slim model of length 20 for CTCF takes approx. 1700 min in the original challenge implementation, the more efficient Catchitt implementation only needs 240 min. Runtimes measured on an Intel Xeon E5-2680v3 using 8 cores for parallel jobs.

Supplementary Methods

Text S1 – Tools for predicting in-vivo binding regions

Most approaches (e.g., [1, 2, 5, 7, 16]) use binding motifs represented as position weight matrix (PWM) models that have been obtained from databases like TRANSFAC [18], Jaspar [19], UniProbe [20] or CisBP [21], or from motif collections like Factorbook [22], the ENCODE-motif collection [23], or Homer [24], while some perform de-novo motif discovery based on k-mers [3] or as part of convolutional neural networks [12, 14]. Irrespective of the source of the motifs considered, three general schemas are have been established for combining motif predictions with chromatin accessibility data. First, motif matches (i.e., predicted binding sites) may be used as prior information and combined with DNase-seq data to distinguish functional from non-functional binding sites (e.g., [1, 11, 8]), Second, TF footprints may be first identified from DNase-seq data and then annotated with specific TFs based on motif matches afterwards [7]. Third, both sources of information are combined in a holistic approach [12, 14]. DNase-seq (and ATAC-seq) data are employed in different ways by existing approaches including i) binning of chromatin accessibility statistics in larger genomic regions around putative binding sites [4], ii) association of chromatin accessibility with specific genes [15], or iii) high-resolution maps of DNase cut sites [6, 8], which may additionally be considered separately for each DNA strand [5]. On the methodological level, approaches either follow a supervised approach based on training examples labeled as "bound" or "unbound", typically derived from TF ChIP-seq data (e.g., [3, 4, 9, 13]), or an unsupervised approach clustering regions into "bound" and "unbound" based on their experimental properties (e.g., DNase-seq data or histone modifications [1, 6, 7]), while others base their predictions on statistical tests [5] or scores related to binding affinity predictions [15]. Supervised approaches use a variety of methods like support vector machines [3, 10], (sparse) logistic regression [2, 4, 9, 16], random forests [13], or neural networks adapted by deep learning [12, 14]. Unsupervised approaches use hierarchical mixture models [1], hierarchical multi-scale models [8], hidden Markov models [7], or other probabilistic models [6]. In some approaches, sequence-based features besides motif matches [10, 7, 16], sequence conservation [10, 13, 16], or additional experimental data like histone modification [1, 3, 7] are included into the model. Finally, a subset of approaches uses the prediction of TF binding regions as an intermediate step for predicting gene regulation [2] or tissue-specific gene expression [15].

Text S2 – Features

The features described in the following are all determined on the level of genome bins. We refer to the bin for which the a-posteriori probability of being peak center should be computed (i.e., the bin containing the peak summit in case of positive examples) as *center bin*. Further, adjacent bins considered are defined relative to that center bin (see also main text, section *Prediction schema*).

S2.1 Sequence-based features

As a first sequence-based feature, we consider the raw DNA sequence according to the *hg19* human genome sequence in the center bin and the directly preceding and the directly following bin. In total, this corresponds to 150 bp of sequence, centered at the center bin.

We further consider the mean G/C-content, and the relative frequency of CG di-nucleotides in the raw sequence spanning those three bins centered at the center bin. G/C-content might be an informative property of promoters bound by a certain TF, and an enrichment of CG di-nucleotides might be informative about the presence of CpG islands.

We also compute the Kullback-Leibler divergence between the relative frequencies of all tri-nucleotides in each of these three bins compared with their relative frequencies in the complete genome. As a feature, we then consider the maximum of those three Kullback-Leibler divergence values obtained for the three bins. Here, the reasoning is that a deviation from the genomic distribution of tri-nucleotides might be a sign of the general information content of a sequence, which might help to distinguish coding and non-coding DNA regions as well as identifying regions that encode regulatory information.

Finally, we consider the length of the longest poly-A or poly-T tract, the length of the longest poly-C or poly-G tract, the length of the longest poly-A/T tract, and the length of the longest poly-G/C tract in these three bins.

All of those sequence-based features are neither TF-specific nor cell type-specific, but model parameters learned on their feature values might well be different for different training TFs or cell types.

S2.2 Annotation-based features

Based on the Gencode v19 genome annotation of the hg19 genome, we derive a set of annotation-based features. First, we consider the distance of the current center bin to the closest TSS annotation (regardless of its strand orientation), which might be informative about core promoter regions. Second, we collect the binary information if the current center bin overlaps with annotations of i) a CDS, ii) a UTR, iii) an exon, iv) a transcript, or v) a TSS annotation, separately for each of the two possible strand orientations. Like some of the previous features, this helps to identify coding, non-coding but transcribed, core promoter, and intergenic regions. Again, these features are not TF or cell type-specific, but model parameters may be adapted specifically for a TF or cell type.

S2.3 Motif-based features

As it might be expected that binding motifs are pivotal for predicting TF-specific binding regions, we create a large collection of motifs for each of the TFs considered. For each of the TFs, we collect all position weight matrix models from the HOCOMOCO database [25] as well as our in-house database DBcorrDB [26], and Slim/LSlim models of the respective TFs from a previous publication [27]. In addition, we learn a large set of motifs from the data provided in the challenge using our motif discovery tools

Dimont [28] using PWM as well as LSlim(3) models [27]. Specifically, we perform motif discovery for

- PWM models from the "conservative" peak files for each training cell type,
- PWM models from the "relaxed" peak files complemented by negative regions selected to be DNase positive (i.e., open chromatin) but ChIP-seq negative according to the ChIP-seq and DNase-seq peak files provided with the challenge data,
- LSlim(3) models from the "conservative" peak files for each training cell type,
- LSlim(3) models from the "relaxed" peak files for each training cell type,
- LSlim(3) models from the "relaxed" peak files complemented by negative regions selected to be DNase positive (i.e., open chromatin) but ChIP-seq negative according to the ChIP-seq and DNase-seq peak files provided with the challenge data.

LSlim(3) may capture intra-motif dependencies between binding site position with a distance of at most three nucleotides.

Motifs discovered using models of different complexity on these different sets of training data ("conservative" and "relaxed" peaks, and "relaxed" peaks complemented by DNase positive regions) should i) capture the breadth of the binding landscape of a TF as represented by the different levels of stringency ("conservative" vs. "relaxed"), and ii) represent potential intra-motif dependencies as well as traditional, "additive" binding affinities. In addition, we learn motifs from the DNase-seq peak files as well, considering

- LSlim(3) models from the "conservative" and "relaxed" DNase-seq peak files,
- LSlim(3) models from the regions in the intersection of all "relaxed" DNase-seq peak files.

Learning motifs from the DNase-seq data alone might have the potential to capture additional binding motifs of TFs that are important for cell type-specific predictions but are not represented in the ChIP-seq data provided with the challenge data.

Regardless of the TF considered, we further include PWM and Slim/LSlim motifs discovered previously [27, 26] for CTCF, SP1, JUND, and MAX, as those i) mark boundaries between regulatory regions, ii) frequently interact with other transcriptions factor, or iii) bind to a large fraction of active promoters. Further TFs that might interact with the currently considered TF as determined i) from the literature, specifically from Factorbook [22], ii) determined from the overlap between the ChIP-seq peaks provided with the challenge data. The latter is accomplished by computing for each TF and cell type i) the TF with the largest overlap (F1 measure computed on the peaks) and ii) the TF with the lowest overlap between the peak files. The former might be indicative of co-binding, while the latter might indicate mutually exclusive binding, both of which might help to predict TF-specific binding regions.

Finally, we consider motifs determined by the epigram pipeline [29], which mark epigenetic modifications. Specifically, we select the top 10 motifs reported for "single mark" analyses for methylation, and H3K4me3 and H3K27ac histone modifications (downloaded from `http://wanglab.ucsd.edu/star/epigram/mods/index.html`).

We use all motif models described above to scan the hg19 genome for potential binding regions. To this end, we apply a sliding window approach across the genome, and aggregate the motif scores obtained according to the genomic bins. For the TF-specific motifs obtained by de-novo motif discovery from ChIP-seq data, we consider as features

- the maximum log-probability of all sliding windows starting in the center bin,
- the logarithm of the sum of binding probabilities in all sliding windows starting in the center bin or its two adjacent bins, and
- the logarithm of the sum of binding probabilities in all sliding windows starting in any of the bins considered.

The first feature should capture the binding affinity at the strongest binding site around the peak summit, while the latter two features represent the general binding affinity of a region with different levels of resolution.

For all of the remaining motifs, we consider the maximum of the bin-wise logarithm of the sum of binding probabilities over all bins considered (see main text, section *Binning the genome*), as this reduces memory requirements as well as model complexity and this level of detail might be sufficient to capture TF interactions.

S2.4 DNase-based features

For the DNase-seq data, the challenge provided tracks with a "fold-enrichment coverage" track, peak files, and the original BAM files from mapping the DNase-seq reads, of which we consider only the former two. From the fold-enrichment coverage track, we compute the following statistics:

- the minimum value across the center bin and its two adjacent bins,
- the minimum of the maximum value within each bin considered,
- the minimum of the 25% percentile within each bin considered, and
- the median values of all the bins considered.

After extracting those feature values for all genomic bins, we quantile normalize each of the features independently across the challenge cell types. Before normalization, we randomize the order of values to avoid systematic effects due to genomic order, which might especially occur for the large number of very low values. For the additional, primary cell types, we do not perform an independent quantile normalization but instead map the DNase-seq features (according to their numerical order) to the corresponding, quantile normalized values of the challenge cell types.

In addition to these short-range DNase features, we also determine a set of long-range features, which are computed from i) 10 bins ii) 20 bins, and iii) 40 bins preceding and succeeding the current center bin. These features are

- the minimum value across all bins,
- the maximum value across all bins,

- the minimum value across the bins preceding the center bin,
- the minimum value across the bins succeeding the center bin,
- the maximum value across the bins preceding the center bin, and
- the maximum value across the bins succeeding the center bin.

Together, these features capture chromatin accessibility on a short and long range level with reasonable resolution, which should be highly informative with regard to the general TF-binding potential. Model parameters should then be able to adapt for TF-specific preferences of chromatin accessibility.

For the current center bin, we additionally determine features of stability across the different cell types, namely

- the ratio of the minimum value in the current cell type divided by the average of the minimum values across all cell types,
- the ratio of the maximum value in the current cell type divided by the average of the maximum values across all cell types,
- the coefficient of variation (standard deviation divided by mean) of the minimum values across all cell types, and
- the coefficient of variation of the maximum values across all cell types,

where the latter two features are identical for all cell types by design.

We also determine several features that represent the monotonicity/stability of these DNase-seq signals. Specifically, these features are

- the number of steps (increasing or decreasing) in the track profile in a 450 bp interval centered at the center bin,
- the longest strictly monotonically increasing stretch in the four bins preceding the center bin,
- the longest strictly monotonically decreasing stretch in the four bins preceding the center bin,
- the longest strictly monotonically increasing stretch in the four bins succeeding the center bin, and
- the longest strictly monotonically decreasing stretch in the four bins succeeding the center bin.

The first of these features has been inspired by the “orange” feature coined by team autosome.ru in the challenge.

Finally, we define further features based on the “conservative” and “relaxed” DNase-seq peak files as provided with the challenge data. These are

- the distance of the center bin to the summit of the closest conservative peak,

- the distance of the center bin to the summit of the closest relaxed peak,
- the peak statistic of a conservative peak overlapping the center bin (or zero if no such overlapping peak exists) multiplied by the length of the overlap,
- the peak statistic of a relaxed peak overlapping the center bin (or zero if no such overlapping peak exists) multiplied by the length of the overlap,
- the maximum of the q-values of an overlapping conservative peak (or zero if no such overlapping peak exists) multiplied by the length of the overlap across the five central bins,
- the maximum of the q-values of an overlapping relaxed peak (or zero if no such overlapping peak exists) multiplied by the length of the overlap across the five central bins.

S2.5 RNA-seq-based features

The RNA-seq data provided with the challenge data included the TPM values of genes according to the gencode v19 genome annotation. TPM values are also quantile normalized across the cell types. As features, we consider

- the maximum TPM value (averaged over the two bio-replicates per cell type) of genes in at most 2.5 kb distance
- the coefficient of variation of the bio-replicated of the corresponding gene,
- the relative difference (difference of values in bio-replicated divided by their mean value) of the corresponding gene.

In analogy to the DNase-based features, we computed from the first feature as measures of stability across the different cell types

- the ratio of the maximum TPM value in the current cell type divided by the average of the maximum values across all cell types, and
- the coefficient of variation of the maximum TPM values across all cell types.

Text S3 – Model & learning principle

For numerical features x, we use independent Gaussian densities parameterized as

$$\mathcal{N}(x|\lambda,\mu) := \sqrt{\frac{e^{\lambda}}{2\pi}} \cdot e^{-\frac{e^{\lambda}}{2}(x-\mu)^2},$$

which allows for unconstrained numerical optimization of both, λ and μ.

For features y with K possible discrete values $v_1,\ldots,v_K$, we use (unnormalized) multinomial distributions with parameters $\boldsymbol{\beta} = (\beta_1,\ldots,\beta_K)$ defined as

$$\mathcal{B}(y|\boldsymbol{\beta}) := \prod_{k=1}^{K} \left(\frac{exp(\beta_k)}{\sum_\ell exp(\beta_\ell)}\right)^{\delta(y=v_k)}.$$

The multinomial coefficient is neglected in this case, since it only depends on the input data but not on the model parameters. In case of binary features, i.e., K=2, this corresponds to an (unnormalized) binomial distribution.

For modeling the raw sequence $\boldsymbol{s} = s_1 s_2 \ldots s_L$, $s_\ell \in \Sigma = \{A, C, G, T\}$, we use a homogeneous Markov model of order 3 parameterized as

$$\mathcal{M}(\boldsymbol{s}|\boldsymbol{\beta}_s) := \frac{exp(\beta_{1,s_1})}{\sum_{a\in\Sigma} exp(\beta_{1,a})} \cdot \frac{exp(\beta_{2,s_2|s_1})}{\sum_{a\in\Sigma} exp(\beta_{2,a|s_1})} \cdot \frac{exp(\beta_{3,s_3|s_1 s_2})}{\sum_{a\in\Sigma} exp(\beta_{3,a|s_1 s_2})} \cdot \prod_{\ell=4}^{L} \frac{exp(\beta_{h,s_\ell|s_{\ell-3}s_{\ell-2}s_{\ell-1}})}{\sum_{a\in\Sigma} exp(\beta_{h,a|s_{\ell-3}s_{\ell-2}s_{\ell-1}})},$$

where $\beta_{h,a|\boldsymbol{b}}, a \in \Sigma, \boldsymbol{b} \in \Sigma^3$ are the homogeneous parameters and $\boldsymbol{\beta}_s = (\beta_{1,A}, \ldots, \beta_{1,T}, \beta_{2,A|A}, \ldots, \beta_{2,T|T}, \beta_{3,A|AA}, \ldots, \beta_{3,T|TT}, \beta_{h,A|AAA}, \ldots, \beta_{h,T|TTT})$ denotes the vector of all model parameters.

Let $\boldsymbol{x} = (x_1, \ldots, x_N)$ denote the vector of all numerical features, $\boldsymbol{y} = (y_1, \ldots, y_M)$ denote the vector of all discrete features, and $\boldsymbol{s}$ denote the raw sequence of one region represented by its feature values $\boldsymbol{z} = (\boldsymbol{x}, \boldsymbol{y}, \boldsymbol{s})$. Let $\boldsymbol{\theta} = (\lambda_1, \ldots, \lambda_N, \mu_1, \ldots, \mu_N, \boldsymbol{\beta}_1, \ldots, \boldsymbol{\beta}_M, \boldsymbol{\beta}_s)$ denote the set of all model parameters. We compute the likelihood of $\boldsymbol{z}$ as an independent product of the terms for the individual features, i.e.,

$$P(\boldsymbol{z}|\boldsymbol{\theta}) := \left(\prod_{\ell=1}^{N} \mathcal{N}(x_\ell|\lambda_\ell, \mu_\ell)\right) \cdot \left(\prod_{\ell=1}^{M} \mathcal{B}(y_\ell|\boldsymbol{\beta}_\ell)\right) \cdot \mathcal{M}(\boldsymbol{s}|\boldsymbol{\beta}_s).$$

For modeling the distribution in the positive (foreground) and negative (background) class, we use likelihoods $P(\boldsymbol{z}|\boldsymbol{\theta}_{fg})$ and $P(\boldsymbol{z}|\boldsymbol{\theta}_{bg})$ with independent sets of parameters $\boldsymbol{\theta}_{fg}$ and $\boldsymbol{\theta}_{bg}$, respectively. In addition, we define the a-priori class probabilities as $P(fg|\gamma_1, \gamma_2) := \frac{exp(\gamma_1)}{exp(\gamma_1)+exp(\gamma_2)}$ and $P(bg|\gamma_1, \gamma_2) = \frac{exp(\gamma_2)}{exp(\gamma_1)+exp(\gamma_2)}$.

Based on these definitions, we may compute the a-posteriori class probability of the positive class as

$$P(fg|\boldsymbol{z}, \boldsymbol{\theta}_{fg}, \boldsymbol{\theta}_{bg}, \boldsymbol{\gamma}) = \frac{P(fg|\gamma_1, \gamma_2) \cdot P(\boldsymbol{z}|\boldsymbol{\theta}_{fg})}{P(fg|\gamma_1, \gamma_2) \cdot P(\boldsymbol{z}|\boldsymbol{\theta}_{fg}) + P(bg|\gamma_1, \gamma_2) \cdot P(\boldsymbol{z}|\boldsymbol{\theta}_{bg})},$$

and the a-posteriori class probability of the negative class in complete analogy.

Using the discriminative maximum conditional likelihood principle [30], the parameters are optimized such that the a-posteriori probabilities of the correct class labels given data and parameters are maximized. Here, we use a variant [31] of the maximum conditional likelihood principle that incorporates weights. Let $\boldsymbol{F} = (\boldsymbol{z}_1, \ldots, \boldsymbol{z}_I)$ denote the set of positive examples and let $\boldsymbol{B} = (\boldsymbol{z}_{I+1}, \ldots, \boldsymbol{z}_J)$ denote the set of negative examples, where $\boldsymbol{z}_i$ is assigned weight w_i. The parameters are then optimized with regard to

$$(\boldsymbol{\theta}^*_{fg}, \boldsymbol{\theta}^*_{bg}, \boldsymbol{\gamma}^*) = \underset{(\boldsymbol{\theta}_{fg}, \boldsymbol{\theta}_{bg}, \boldsymbol{\gamma})}{\operatorname{argmax}} \left[\sum_{i=1}^{I} w_i \cdot \log P(fg|\boldsymbol{z}_i, \boldsymbol{\theta}_{fg}, \boldsymbol{\theta}_{bg}, \boldsymbol{\gamma}) + \sum_{i=I+1}^{J} w_i \cdot \log P(bg|\boldsymbol{z}_i, \boldsymbol{\theta}_{fg}, \boldsymbol{\theta}_{bg}, \boldsymbol{\gamma}) \right].$$

Text S4 – Sampling of DNase-matched negative regions

We sample negative regions with chromatin accessibility values matched to the positive regions (following an idea related to importance sampling) as explained in the following. We consider the center bins of all positive regions, collect the corresponding DNase-seq median feature values (see Text S2) of those bins, and determine a histogram of the collected values. The histogram is composed of 20 equally sizes bins between the observed maximum and minimum values of the DNase-seq median values. This histograms represents an approximation of the distribution of DNase-seq median values in the positive regions. As we expect DNase-seq values to be highly informative about TF binding, we aim at sampling a representative set of negative regions that exhibit similar DNase-seq values but might be distinguished from positive regions by other features.

To this end, we assign each of the negative regions to the same histogram bins based on their respective DNase-seq median values at their center bins. This also yields an analogous histogram of the DNase-seq median values for the negative regions, which will usually be different from the histogram for the positive regions.

Within each histogram bin, we then draw a subset of the negative regions assigned to that bin by i) drawing a subset of these regions four times as large as the corresponding positive set, and ii) weighting the drawn negative regions such that the sum of weights matches the relative abundance of that histogram bin in the histogram on all negative region.

Conceptually, this procedure yields an over-sampling of negative regions with large DNase-seq median features, which is adjusted for by down-weighting such examples to the corresponding frequency on the chromosome level. This is especially important as these will be regions that are hard to classify using DNase-seq based features but are only lowly represented by the uniform sampling schema.

Text S5 – Implementation notes

The code for model training and prediction of both the challenge implementation and also the Catchitt implementation is based in the Java library Jstacs [32] available from github (`https://github.com/Jstacs/Jstacs`). In the following, we list specific classes that have been used in either or both of the implementations. The classes specific to the challenge implementation realizing, for instance, the iterative training procedure can be found in package `projects.dream2016`, while the Catchitt implementation can be found in package `projects.encodedream`.

The product of Gaussian densities used in both versions of the code is implemented in the class `de.jstacs.sequenceScores.statisticalModels.differentiable.continuous.GaussianNetwork`. While this class generally supports Gaussian networks including dependencies between random variables, we handle features independently (as specified by the `structure` parameter) in this case.

For discrete features, which are included only in the challenge implementation, we use the class `de.jstacs.sequenceScores.statisticalModels.differentiable.directedGraphicalModels.BayesianNetworkDiffSM` implementing fixed-structure discrete Bayesian networks including inhomogeneous Markov models, and the class `de.jstacs.sequenceScores.statisticalModels.differentiable.homogeneous.HomogeneousMMDiffSM` for homogeneous Markov models.

Models for the individual labels on the training data (i.e., classes in the sense of classification problems) are wrapped in a generic classifier class `de.jstacs.classifiers.differentiableSequenceScoreBased.gendismix.GenDisMixClassifier` using the discriminative maximum conditional likelihood principle for parameter estimation. As parameter estimation needs to be carried out numerically, this class uses an implementation of a quasi-Newton optimization method, internally, as implemented in `de.jstacs.algorithms.optimization.Optimizer` (method `quasiNewtonBFGS`).

Core classes for the iterative training procedures used in Catchitt and in the challenge implementation are `projects.encodedream.IterativeTraining` and `projects.dream2016.IterativeTraining_Round2`, respectively.

After model (and classifier) parameters have been adapted on the training data, binding probabilities for individual feature vectors from the test data are determined using the `getScores` method of `de.jstacs.classifiers.differentiableSequenceScoreBased.gendismix.GenDisMixClassifier`.

Averaging over classifier predictions is performed in the method `aggregate` of `projects.encodedream.Predictor` and class `projects.dream2016.Aggregation_multi`, respectively.

References

[1] Pique-Regi, R., Degner, J.F., Pai, A.A., Gaffney, D.J., Gilad, Y., Pritchard, J.K.: Accurate inference of transcription factor binding from DNA sequence and chromatin accessibility data. Genome Research **21**(3), 447–455 (2011)

[2] Natarajan, A., Yardimci, G.G., Sheffield, N.C., Crawford, G.E., Ohler, U.: Predicting cell-type–specific gene expression from regions of open chromatin. Genome Research **22**(9), 1711–1722 (2012)

[3] Arvey, A., Agius, P., Noble, W.S., Leslie, C.: Sequence and chromatin determinants of cell-type–specific transcription factor binding. Genome Research **22**(9), 1723–1734 (2012)

[4] Luo, K., Hartemink, A.J.: Using DNase digestion data to accurately identify transcription factor binding sites. In: Pacific Symposium on Biocomputing, pp. 80–91. World Scientific, Singapore (2012)

[5] Piper, J., Elze, M.C., Cauchy, P., Cockerill, P.N., Bonifer, C., Ott, S.: Wellington: a novel method for the accurate identification of digital genomic footprints from DNase-seq data. Nucleic Acids Research **41**(21), 201 (2013)

[6] Sherwood, R.I., Hashimoto, T., O'Donnell, C.W., Lewis, S., Barkal, A.A., van Hoff, J.P., Karun, V., Jaakkola, T., Gifford, D.K.: Discovery of directional and nondirectional pioneer transcription factors by modeling DNase profile magnitude and shape. Nat Biotech **32**(2), 171–178 (2014)

[7] Gusmao, E.G., Dieterich, C., Zenke, M., Costa, I.G.: Detection of active transcription factor binding sites with the combination of DNase hypersensitivity and histone modifications. Bioinformatics **30**(22), 3143–3151 (2014)

[8] Raj, A., Shim, H., Gilad, Y., Pritchard, J.K., Stephens, M.: msCentipede: Modeling heterogeneity across genomic sites and replicates improves accuracy in the inference of transcription factor binding. PLOS ONE **10**(9), 1–15 (2015)

[9] Kähärä, J., Lähdesmäki, H.: BinDNase: a discriminatory approach for transcription factor binding prediction using DNase I hypersensitivity data. Bioinformatics **31**(17), 2852–2859 (2015)

[10] Kumar, S., Bucher, P.: Predicting transcription factor site occupancy using DNA sequence intrinsic and cell-type specific chromatin features. BMC Bioinformatics **17**(1), 4 (2016)

[11] Jankowski, A., Tiuryn, J., Prabhakar, S.: Romulus: robust multi-state identification of transcription factor binding sites from DNase-seq data. Bioinformatics **32**(16), 2419–2426 (2016)

[12] Quang, D., Xie, X.: FactorNet: a deep learning framework for predicting cell type specific transcription factor binding from nucleotide-resolution sequential data. bioRxiv (2017). doi:10.1101/151274

[13] Liu, S., Zibetti, C., Wan, J., Wang, G., Blackshaw, S., Qian, J.: Assessing the model transferability for prediction of transcription factor binding sites based on chromatin accessibility. BMC Bioinformatics **18**(1), 355 (2017)

[14] Qin, Q., Feng, J.: Imputation for transcription factor binding predictions based on deep learning. PLOS Computational Biology **13**(2), 1–20 (2017)

[15] Schmidt, F., Gasparoni, N., Gasparoni, G., Gianmoena, K., Cadenas, C., Polansky, J.K., Ebert, P., Nordström, K., Barann, M., Sinha, A., Fröhler, S., Xiong, J., Dehghani Amirabad, A., Behjati Ardakani, F., Hutter, B., Zipprich, G., Felder, B., Eils, J., Brors, B., Chen, W., Hengstler, J.G., Hamann, A., Lengauer, T., Rosenstiel, P., Walter, J., Schulz, M.H.: Combining transcription factor binding affinities with open-chromatin data for accurate gene expression prediction. Nucleic Acids Research **45**(1), 54–66 (2017)

[16] Chen, X., Yu, B., Carriero, N., Silva, C., Bonneau, R.: Mocap: large-scale inference of transcription factor binding sites from chromatin accessibility. Nucleic Acids Research **45**(8), 4315–4329 (2017)

[17] Kharchenko, P.V., Tolstorukov, M.Y., Park, P.J.: Design and analysis of ChIP-seq experiments for DNA-binding proteins. Nat Biotech **26**(12), 1351–1359 (2008)

[18] Matys, V., Kel-Margoulis, O.V., Fricke, E., Liebich, I., Land, S., Barre-Dirrie, A., Reuter, I., Chekmenev, D., Krull, M., Hornischer, K., Voss, N., Stegmaier, P., Lewicki-Potapov, B., Saxel, H., Kel, A.E., Wingender, E.: TRANSFAC(R) and its module TRANSCompel(R): transcriptional gene regulation in eukaryotes. Nucleic Acids Research **34**(suppl_1), 108–110 (2006)

[19] Mathelier, A., Fornes, O., Arenillas, D.J., Chen, C.-y., Denay, G., Lee, J., Shi, W., Shyr, C., Tan, G., Worsley-Hunt, R., Zhang, A.W., Parcy, F., Lenhard, B., Sandelin, A., Wasserman, W.W.: Jaspar 2016: a major expansion and update of the open-access database of transcription factor binding profiles. Nucleic Acids Research **44**(D1), 110–115 (2016)

[20] Newburger, D.E., Bulyk, M.L.: UniPROBE: an online database of protein binding microarray data on protein-DNA interactions. Nucleic Acids Research **37**(suppl 1), 77–82 (2009)

[21] Weirauch, M.T., Yang, A., Albu, M., Cote, A.G., Montenegro-Montero, A., Drewe, P., Najafabadi, H.S., Lambert, S.A., Mann, I., Cook, K., Zheng, H., Goity, A., van Bakel, H., Lozano, J.-C., Galli, M., Lewsey, M.G., Huang, E., Mukherjee, T., Chen, X., Reece-Hoyes, J.S., Govindarajan, S., Shaulsky, G., Walhout, A.J.M., Bouget, F.-Y., Ratsch, G., Larrondo, L.F., Ecker, J.R., Hughes, T.R.: Determination and inference of eukaryotic transcription factor sequence specificity. Cell **158**(6), 1431–1443 (2014)

[22] Wang, J., Zhuang, J., Iyer, S., Lin, X., Whitfield, T.W., Greven, M.C., Pierce, B.G., Dong, X., Kundaje, A., Cheng, Y., Rando, O.J., Birney, E., Myers, R.M., Noble, W.S., Snyder, M., Weng, Z.: Sequence features and chromatin structure around

the genomic regions bound by 119 human transcription factors. Genome Research **22**(9), 1798–1812 (2012)

[23] Kheradpour, P., Kellis, M.: Systematic discovery and characterization of regulatory motifs in ENCODE TF binding experiments. Nucleic Acids Research **42**(5), 2976–2987 (2014)

[24] Heinz, S., Benner, C., Spann, N., Bertolino, E., Lin, Y.C., Laslo, P., Cheng, J.X., Murre, C., Singh, H., Glass, C.K.: Simple combinations of lineage-determining transcription factors prime cis-regulatory elements required for macrophage and B cell identities. Molecular Cell **38**(4), 576–589 (2010)

[25] Kulakovskiy, I.V., Vorontsov, I.E., Yevshin, I.S., Soboleva, A.V., Kasianov, A.S., Ashoor, H., Ba-alawi, W., Bajic, V.B., Medvedeva, Y.A., Kolpakov, F.A., Makeev, V.J.: HOCOMOCO: expansion and enhancement of the collection of transcription factor binding sites models. Nucleic Acids Research **44**(D1), 116–125 (2016)

[26] Grau, J., Grosse, I., Posch, S., Keilwagen, J.: Motif clustering with implications for transcription factor interactions. In: German Conference on Bioinformatics. PeerJ Preprints, vol. 3, p. 1601 (2015)

[27] Keilwagen, J., Grau, J.: Varying levels of complexity in transcription factor binding motifs. Nucleic Acids Research (2015)

[28] Grau, J., Posch, S., Grosse, I., Keilwagen, J.: A general approach for discriminative de novo motif discovery from high-throughput data. Nucleic Acids Research **41**(21), 197 (2013)

[29] Whitaker, J.W., Chen, Z., Wang, W.: Predicting the human epigenome from DNA motifs. Nat Meth **12**(3), 265–272 (2015)

[30] Roos, T., Wettig, H., Grünwald, P., Myllymäki, P., Tirri, H.: On discriminative Bayesian network classifiers and logistic regression. Machine Learning **59**(3), 267–296 (2005)

[31] Grau, J.: Discriminative Bayesian principles for predicting sequence signals of gene regulation. PhD thesis, Martin Luther University Halle–Wittenberg (April 2010)

[32] Grau, J., Keilwagen, J., Gohr, A., Haldemann, B., Posch, S., Grosse, I.: Jstacs: A Java framework for statistical analysis and classification of biological sequences. Journal of Machine Learning Research **13**(Jun), 1967–1971 (2012)
